# Therapeutic efficacy, pharmacokinetic profiles, and toxicological activities of humanized antibody-drug conjugate Zt/g4-MMAE targeting RON receptor tyrosine kinase for cancer therapy

**DOI:** 10.1186/s40425-019-0525-0

**Published:** 2019-03-14

**Authors:** Hang-Ping Yao, Liang Feng, Sreedhar Reddy Suthe, Ling-Hui Chen, Tian-Hao Weng, Chen-Yu Hu, Eun Sung Jun, Zhi-Gang Wu, Wei-Lin Wang, Song Cheol Kim, Xiang-Min Tong, Ming-Hai Wang

**Affiliations:** 1 State Key Laboratory for Diagnosis & Treatment of Infectious Diseases, First Affiliated Hospital, Zhejiang University School of Medicine, Hangzhou, China; 2 Cancer Biology Research Center, Hangzhou, China; 3 Department of Pharmaceutical Sciences, Texas Tech University Health Sciences Center School of Pharmacy, Amarillo, TX USA; 4 Zhejiang Provincial Key Laboratory for Precision Diagnosis & Treatment of Hepatic & Pancreatic Oncology, Zhejiang Province, China; 5 Division of Hepatobiliary & Pancreatic Surgery, First Affiliated Hospital, Zhejiang University School of Medicine, Hangzhou, China; 6 Department of Biomedical Sciences, Kowloon Tong, Hong Kong; 7 Biliary and Pancreatic Cancer Center at Department of Surgery, University of Ulsan College of Medicine, Seoul, South Korea; 8 Department of Laboratory Medicine, Zhejiang Provincial People’s Hospital, Hangzhou Medical College, Hangzhou, China

**Keywords:** Pancreatic cancer, RON receptor tyrosine kinase, Antibody-rug conjugates, Pharmacokinetics, Xenograft tumor model, Therapeutic efficacy, Toxicological profiles

## Abstract

**Background:**

Aberrant expression of the RON receptor tyrosine kinase is a pathogenic feature and a validated drug target in various types of cancers. Currently, therapeutic antibodies targeting RON for cancer therapy are under intensive evaluation. Here we report the development and validation of a novel humanized anti-RON antibody-drug conjugate for cancer therapy.

**Methods:**

Antibody humanization was achieved by grafting sequences of complementarity-determining regions from mouse monoclonal antibody Zt/g4 into human IgG1/κ acceptor frameworks. The selected humanized Zt/g4 subclone H1L3 was conjugated with monomethyl auristatin E using a dipeptide linker to form H-Zt/g4-MMAE. Pharmacokinetic analysis of H-Zt/g4-MMAE was determined using hydrophobic interaction chromatography and a MMAE ADC ELISA kit. Biochemical and biological assays were used for measuring RON expression, internalization, cell viability and death. Therapeutic efficacies of H-Zt/g4-MMAE were validated in vivo using three pancreatic cancer xenograft models. Toxicological activities of H-Zt/g4-MMAE were determined in mouse and cynomolgus monkey.

**Results:**

H-Zt/g4-MMAE had a drug to antibody ratio of 3.77:1 and was highly stable in human plasma with a dissociation rate less than 5% within a 20 day period. H-Zt/g4-MMAE displayed a favorable pharmacokinetic profile in both mouse and cynomolgus monkey. In vitro, H-Zt/g4-MMAE induced RON internalization, which results in killing of pancreatic cancer cells with IC_50_ values at 10–20 nM. In vivo*,* H-Zt/g4-MMAE inhibited pancreatic cancer xenograft growth with tumoristatic concentrations at 1~3 mg/kg bodyweight. Significantly, H-Zt/g4-MMAE eradicated tumors across multiple xenograft models regardless their chemoresistant and metastatic statuses. Moreover, H-Zt/g4-MMAE inhibited and eradicated xenografts mediated by pancreatic cancer stem-like cells and by primary cells from patient-derived tumors. Toxicologically, H-Zt/g4-MMAE is well tolerated in mice up to 60 mg/kg. In cynomolgus monkey, H-Zt/g4-MMAE up to 30 mg/kg had a manageable and reversible toxicity profile.

**Conclusions:**

H-Zt/g4-MMAE is superior in eradication of pancreatic cancer xenografts with favorable pharmacokinetic profiles and manageable toxicological activities. These findings warrant the transition of H-Zt/g4-MMAE into clinical trials in the future.

**Electronic supplementary material:**

The online version of this article (10.1186/s40425-019-0525-0) contains supplementary material, which is available to authorized users.

## Background

The RON receptor tyrosine kinase [1], a member of the MET proto-oncogene family [2], is implicated in pathogenesis of various cancers including those from breast, colon, lung, and pancreas [3]. Accumulated evidences indicate that RON is overexpressed in a significant portion in epithelial cancers [4–10]. In pancreatic ductal adenocarcinoma (PDAC), RON is overexpressed in more than 35% of primary tumor samples [4, 5, 9] and associated with tumor progression [4, 5]. Increased RON expression also serves as an indicator for the shortened survival of breast cancer patients [7]. At the cellular level, aberrant RON expression is associated with production of various truncated or splicing variants [8, 11–13], which exert tumorigenic activities facilitating cancer cell growth, migration, invasion, and chemoresistance [14–17]. In addition, RON overexpression transduces signaling that promotes epithelial to mesenchymal transition leading to an aggressive invasive phenotype [14–18]. These features help not only to establish the role of RON in cancer development, but also to provide the molecular basis of targeting RON for cancer therapy.

The current strategies of targeting RON for cancer therapy focus on small-molecule kinase inhibitors (SMKI) and therapeutic antibodies [19–25]. In preclinical studies, RON-specific SMKI and therapeutic antibodies are effective in killing cancerous cells and in inhibiting xenograft tumors from multiple sources [19–25]. In phase 1 clinical trials in patients with advanced solid tumors, narnatumab, an anti-RON therapeutic antibody, has been found to be well tolerated with limited antitumor activity in the designed dosing regimen [26]. Nevertheless, the lack of strong efficacy prevents narnatumab moving forward for further clinical evaluation. Clearly, improvement in therapeutic efficacy of anti-RON antibody is critically important for clinical application.

Antibody-drug conjugate (ADC) is a therapeutic strategy combining target-specific antibody with highly potent cytotoxic drug for cancer treatment [27, 28]. Since 2014, we have focused our effort on developing anti-RON ADCs with improved therapeutic index for potential cancer therapy [29–33]. By selecting suitable anti-RON monoclonal antibodies (mAb) such as Zt/g4 [34, 35], we have generated the first anti-RON ADC Zt/g4-DM1 (Zt/g4 conjugated with maytansinoid DM1) via thioether linkage technology [29]. Zt/g4-DM1 is effective in inhibition of xenograft tumors derived from colon, breast, and lung cancer cell lines [29–31]. However, the effect of Zt/g4-DM1 on pancreatic xenograft tumors is relatively weak [30]. To improve the efficacy for Zt/g4-based ADCs, we conjugated Zt/g4 with monomethyl auristatin E (MMAE) to generate Zt/g4-MMAE using a protease-sensitive dipeptide linker [31, 32]. Studies in vivo confirmed that Zt/g4-MMAE is highly potent in inhibition and/or eradication of xenograft tumors initiated by PDAC cells [32], which is insensitive to Zt/g4-DM1 [30]. Moreover, Zt/g4-MMAE completely eradicates xenograft tumors initiated by triple-negative breast cancer (TNBC) cell lines [33]. Both cancers are known to be highly malignant with limited treatment options [36, 37]. We conclude from these findings that Zt/g4-MMAE has potentials for clinical development.

The study presented here is our continued effort in development of Zt/g4-MMAE as a lead candidate for potential clinical application. To this end, humanized (H)-Zt/g4 was generated, selected, and conjugated with MMAE to form H-Zt/g4-MMAE. Various in vitro and in vivo experiments were performed to validate H-Zt/g4-MMAE activities for drug delivery, cellular cytotoxicity, and inhibition and/or eradication of PDAC xenografts. Moreover, H-Zt/g4-MMAE pharmacokinetic (PK) profiles and toxicological activities in both mouse and cynomolgus monkey were determined. These studies demonstrated that H-Zt/g4-MMAE is a highly effective anti-RON ADC with manageable toxicological profiles, which lays the foundation for clinical development.

## Materials and methods

### Cell lines, reagents, and animals

PDAC Panc-1 and BxPC-3 and CRC LoVo, HT-29, HCT116, and SW620 cell lines were from American Type Cell Culture (Manassas, VA). Additional PDAC cell lines FG and L3.6pl were provided by Drs. A.M. Lowy (University of California at San Diego, San Diego, CA) and G.E. Gallick (University of Texas M.D. Anderson Cancer Center, Houston, TX), respectively. PDAC stem-like cells expressing CD24, CD44, and epithelial specific antigen (ESA), designated as PSC^+ 24/44/ESA^, were prepared from spheroid cells derived from BxPC-3, FG, and L3.6pl cells as previously described [38, 39]. NIH3T3 cells expressing human, monkey, or mouse RON were generated by transfection of pcDNA3 containing individual RON cDNAs as previously described [8]. Primary PDAC cell lines AMCPAC02, AMCPAC04, SNU2491, and SNU410 established from patient-derived tumors were used as previously described [40]. Mouse anti-RON mAb Zt/g4, Zt/f2 and rabbit IgG antibody against the RON C-terminus were used as previously described [8, 34]. Female athymic nude mice at 6 weeks of age were from Taconic Biosciences (Granbury, NJ). The use of mice was approved by the Texas Tech University institutional animal care committee. Female cynomolgus monkeys aged at 3.6–4 years with an average bodyweight of 2.6 ± 0.25 kg/animal were from Guidong Quadrumana Development & Laboratory (Guangxi, China). The use of animal was approved by the Zhejiang University School of Medicine institutional review committee according to the Chinese Food &Drug administration guidelines.

### Analyses of RON expression, internalization, cell viability, and death

Levels of RON expression by PDAC cells was determined using Z/tg4 in flow cytometric analysis as previously described [30]. H-Zt/g4-induced RON internalization by PDAC cells was determined in immunofluorescence analysis as previously described [31]. PDAC cell viability and cell death after H-Zt/g4-MMAE treatment was determined by the MTS assay and the trypan blue exclusion assay as previously described [30, 31].

### Western blot analysis of RON protein in PDAC xenograft tumors

Lysates from xenograft tumors were prepared in a lysis buffer as previously described [8]. Cellular proteins (20 μg per sample) were separated in an 8% SDS-PAGE under reduced conditions. RON was detected using the rabbit anti-RON IgG antibody [8] with enhanced chemiluminescent reagents and analyzed in Bio-Rad Versa-Doc 5000 Image system. Membranes also were reprobed with antibody specific to actin to ensure equal sample loading.

### Generation of humanized Zt/g4 and antibody-drug conjugates

Antibody humanization was performed by grafting the sequences from complementarity-determining regions (CDRs) of Zt/g4 into human IgG1/κ acceptor frameworks to generate five light and five heavy chains to create 25 different parings of H-Zt/g4 IgG1/κ molecules [41]. The subclone H1L3 was selected as the lead candidate. DM1, MMAE, and duocarmycin were from Concortis (www.concortis.com) and used for conjugation according to the manufacturer’s instruction. H-Zt/g4-MMAE with DAR of 3.77:1 was selected as the lead candidate. Zt/g4-DM1, H-Zt/g4-DM1, and Zt/g4-MMAE also were prepared and used for comparison. Control mouse IgG conjugated with MMAE (CmIgG-MMAE) at a DAR of 4.1:1 served as the control. All ADCs were purified, sterilized through a filter, and verified by hydrophobic interaction chromatography (HIC) [31].

### Analysis of H-Zt/g4-MMAE stability in human plasma in vitro and in cynomolgus monkey in vivo

H-Zt/g4-MMAE at 10 μg per ml was incubated in human fresh plasma in 1 ml plasma at 37 °C up to 20 days. Samples were collected at different time intervals. H-Zt/g4-MMAE in the plasma of cynomolgus monkey (three animals per group) was collected at different time intervals after a single injection of H-Zt/g4-MMAE at 10 or 30 mg/kg. Free MMAE was determined by using the LC-MS/MS method [42] with slight modifications.

### Pharmacokinetic analysis of H-Zt/g4-MMAE in mouse and in cynomolgus monkey

Female nude mice (five mice per group, with or without PDAC xenograft tumors) were injected with a single dose of H-Zt/g4-MMAE at 3, 10, and 20 mg/kg through tail vein. Cynomolgus monkeys (three animals per group) were administered through saphenous vein with a single dose of H-Zt/g4-MMAE at 10 and 30 mg/kg. Blood samples were collected from individual mice or monkeys at different time intervals. The amount of MMAE conjugated H-Zt/g4 in plasma was determined by using a MMAE ADC ELISA kit (Eagle Biosciences Inc., Nashua, NH). The PK parameters were calculated using the WinNonLin software package (Certara, Princeton, NJ) as previously described [30].

### Xenograft PDAC model and H-Zt/g4-MMAE treatment

Female athymic nude mice were injected with 5 × 10^6^ PDAC cells in 0.1 ml PBS into the subcutaneous space of the right flank of mice as previously described [30, 31]. Xenograft tumors mediated by CRC cells served for comparison. For PDAC stem-like cell-mediated xenograft tumors, individual mice were injected with 5 × 10^5^ PSC^+ 24/44/ESA^ cells in 0.1 ml PBS as described above. For PDX models, primary PDAC cells from patient-derived tumors at 5 × 10^6^ cells in 0.1 ml PBS were used. Mice were randomized into different groups (five mice per group). Treatment began when all tumors had a mean volume of ~ 150 mm^3^. Three treatment regimens, H-Zt/g4-MMAE at 1, 3, 7, 10, and 15 mg/kg in a Q6 days × 5 schedule, at 20 mg/kg in a Q12 days × 2 schedule, and at 10 mg/kg in a Q12 × 2 schedule, were used and injected through tail vein in a volume of 0.1 ml PBS. Tumor volumes were measured every four days as previously described [30, 31]. All mice were sacrificed at the end of experiments. Tumors from individual mice were collected and weighted to reach an average for each group. The percentage of inhibition was calculated as previously described [30, 31].

### Toxicological studies of H-Zt/g4-MMAE in mouse and cynomolgus monkey

H-Zt/g4-MMAE at 10 and 30 mg/kg in a single dose or PBS was injected through the saphenous vein into cynomolgus monkeys (three animals per group). Animals were monitored daily for responsiveness, food consumption, bodyweight, temperature, pulse, and others. Urine and blood samples were collected according to the schedule. Electrocardiogram was performed before and after ADC injection every five days. Blood leukocytes and various enzymatic activities were measured as previously described [43]. All animals were sacrificed at the end of the study. Organs and tissues from individual monkeys were collected and weighted. Histological examinations were performed on all organs and tissues.

### Statistical analysis

GraphPad 6 software was used for regular statistical analysis. Results are shown as mean ± SD. The data between control and experimental groups were compared using Student *t* test. The WinNonLin soft package was used for pharmacokinetic analysis. Statistical differences at *p* < 0.05 were considered significant.

## Results

### Generation of H-Zt/g4-MMAE with favorable pharmacological properties

**Fig. 1:**
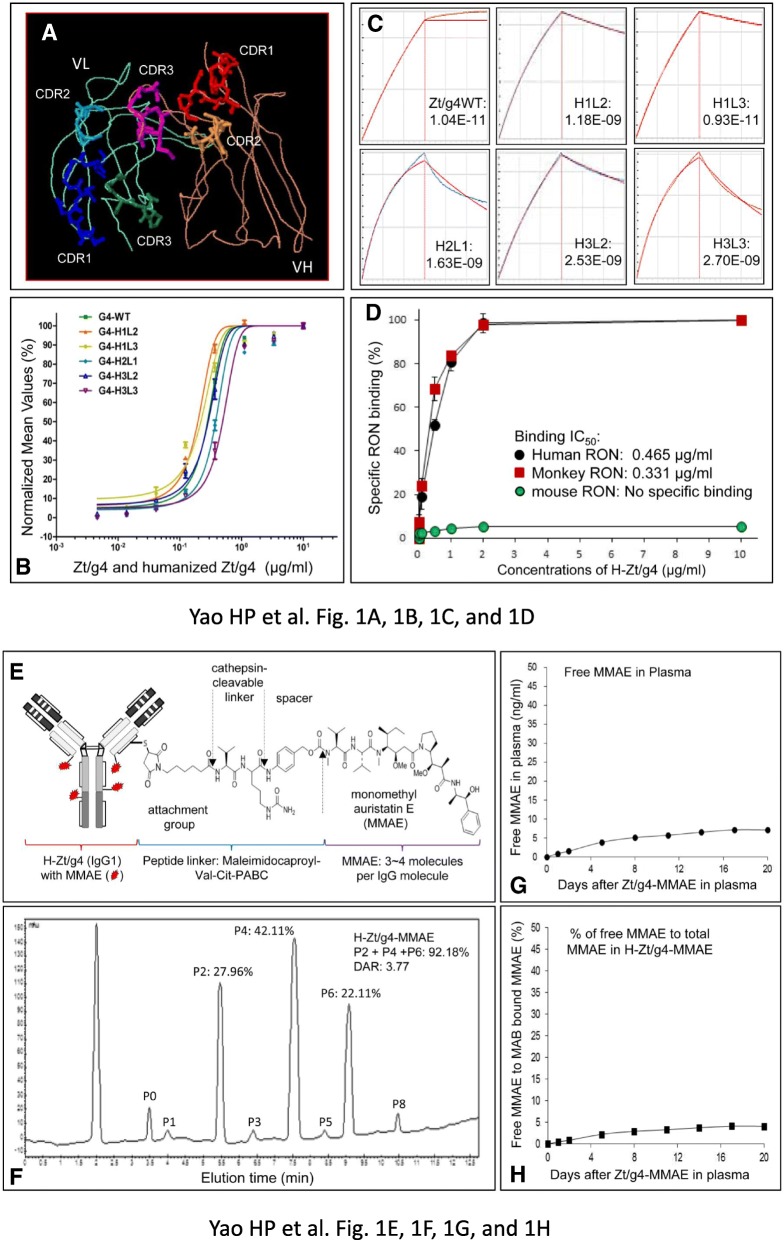
Generation of humanized Zt/g4 antibody and characterization of RON-targeted antibody-drug conjugates: (**a**) Modeling of CDRs from mouse Zt/g4 in the variable regions of human IgG heavy chain and light chain. The framework of human IgG1 molecule was used for Zt/g4 humanization. The models of Zt/g4 CDRs grafted in the variable regions of human IgG1 heavy chain and light chain were generated by using the software PIGS from Automatic Predictions of Immunoglobulin Structures (Tramontano at University of Rome, Italy). (**b**) Binding of subclone H-Zt/g4 molecules to human RON. Different amounts of individual H-Zt/g4 s were incubated with NIH-3 T3 cells expressing human RON followed by addition of goat anti-human IgG1 antibody coupled with FITC. (**c**) Kinetic characterization of H-Zt/g4 interaction with human RON proteins by Octet RED96 system. Pure RON proteins from lysates of NIH3T3 cells expressing RON were immobilized onto the amine reactive sensor and assayed against individual H-Zt/g4 molecules in duplicate. The data set is analyzed with global fitting to produce the antibody-receptor binding affinity (*K*_D_). Blue curves represent experimental data and red curves represent the statistical fitting of curves. (**d**) Interaction of H-Zt/g4 H1L3 with RONs from different species. NIH3T3 cells expressing human, monkey, or mouse RON were incubated with H-Zt/g4 H1L3 followed by goat anti-human IgG coupled with FITC. Immunofluorescent intensities from individual samples were determined by flow cytometric analysis. (**e**) Schematic representation of H-Zt/g4-MMAE structure. MMAE was conjugated to H-Zt/g4 by the valine-citruline dipeptide linker according to the manufacturer’s instruction (www.concortis.com). (**f**) HIC analysis of MMAE conjugated to H-Zt/g4: Individual Zt/g4-MMAEs with different numbers of MMAE (0 to 8) are marked as P0 to P8. A DAR combining P2, P4, and P6 at 3.77:1 was achieved. (**g**) Free MMAE dissociated from H-Zt/g4-MMAE in human plasma. H-Zt/g4-MMAE at 10 μg per ml was incubated with fresh human plasma at 37 °C for 20 days. The amount of free MMAE in plasma was determined using the LC-MS/MS method with slight modifications. (**h**) Samples from (**g**) were used also for measuring MMAE conjugated H-Zt/g4 as detailed in Materials and Methods. A ratio from free MMAE to the total MMAE in H-Zt/g4-MMAE was calculated to determine the percentages of MMAE dissociated from H-Zt/g4-MMAE

Structures of CDRs from Zt/g4 in human IgG1/κ acceptor frameworks is shown in Fig. 1a**.** H-Zt/g4 has a binding affinity of 3.07 nM per ml for human RON (Fig. 1b and c). It also recognized cynomolgus monkey RON with a binding affinity at 2.21 nM per ml (Fig. 1d) but not mouse RON. Zt/g4 immunohistochemical staining of RON in multiple tissues from human or monkey is shown in Additional file 1: Figure S1. RON was detected at low levels in various epithelial cells from digestive, liver, kidney and other epithelial tissues. The patterns of RON reactivity between human and monkey tissues were similar.

The representative structure of H-Zt/g4-MMAE with an average of drug to antibody ratio (DAR) at 3.77:1 was shown in Fig. 1e and f. The conjugation profile fits ADCs formulated using the dipeptide linker Val-Cit-PABC [42]. H-Zt/g4-MMAE is stable in human plasma in vitro with less than 5% of MMAE dissociated from the conjugates after incubation up to 20 days (Fig. 1g and h).

### Induction by H-Zt/g4-MMAE of RON internalization and cellular cytotoxicity

Four PDAC cell lines expressing variable levels of RON were used to evaluate H-Zt/g4-MMAE-induced RON internalization. The calculated RON molecules per PDAC cell were 10,214 ± 310 for BxPC-3, 13,178 ± 269 for FG, and 16,628 ± 245 for L3.6pl cells, respectively. Specific binding was not observed in Panc-1 cells (< 10 ± 2), which served as the control.

**Fig. 2:**
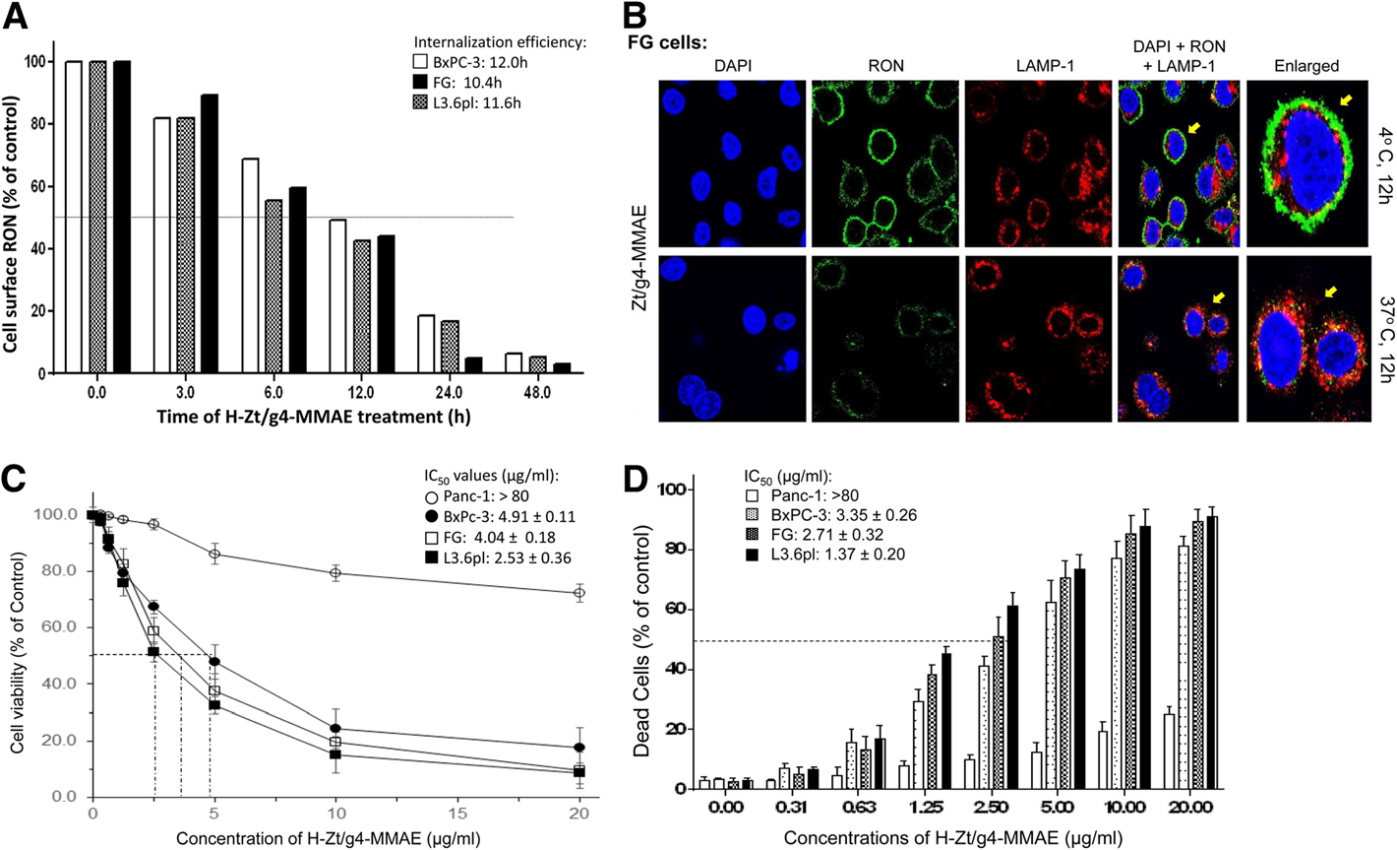
Effect of H-Zt/g4-MMAE on RON internalization, cell viability, and death: (**a**) H-Zt/g4-induced cell surface RON internalization. PDAC cell lines BxPC-3, FG and L3.6pl (1 × 10^6^ cells per dish) were treated at 37 °C with 5 μg/ml of H-Zt/g4-MMAE, collected at different time points, washed with acidic buffer to eliminate cell surface bound IgG [31], and then incubated with 2 μg/mL of anti-RON mAb Zt/c1 [34]. Immunofluorescence was analyzed by flow cytometer using FITC-coupled anti-mouse IgG. Immunofluorescence from cells treated with H-Zt/g4 at 4 °C was set as 100%. Internalization efficiency (IC_50_) was calculated as the time required achieving 50% reduction of cell surface RON. (**b**) Intracellular localization of internalized RON. FG cells in a 6-well plate were treated with 5 μg/ml of H-Zt/g4 at 4 °C or 37 °C for 12 h followed by mouse anti-human IgG1-coupled with FITC. Nuclear DNAs were stained with DAPI. LAMP-1 was used as a marker for protein cytoplasmic localization. Similar results also observed in additional three PDAC cell lines (data not shown). (**c**) Effect of H-Zt/g4-MMAE on viability of PDAC cells. Three PDAC cell lines (8000 cells per well in a 96-well plate in triplicate) were treated with different amounts of H-Zt/g4-MMAE for 96 h. Panc-1 cells served as the negative control. Cell viability was determined by the MTS assay. (**d**) Death of PDAC cells after H-Zt/g4-MMAE treatment. PDAC cells were treated with different amounts of H-Zt/g4-MMAE for 96 h. The percentages of cell death were determined by the trypan blue exclusion method. Data shown in (**c**) and (**d**) are derived from one of three experiments with similar results

We first determined H-Zt/g4-MMAE-induced RON internalization (Fig. 2a). Less than 10% of RON remained on the cell surface 48 h after H-Zt/g4-MMAE treatment. The time required for H-Zt/g4-MMAE to induce a 50% RON reduction (internalization efficacy) was 12.0 h, 10.4 h, and 11.6 h for BxPC-3, FG, and L3.6pl, respectively. Immunofluorescence analysis using FG cells as the model confirmed intracellular RON localization with lysosomal-associated membrane protein (LAMP)-1 **(**Fig. 2b). H-Zt/g4-MMAE induced a significant reduction in PDAC cell viability in a dose-dependent manner (Fig. 2c). The IC_50_ values at 96 h were 4.91 ± 0.11 μg per ml for BxPC-3, 4.04 ± 0.18 μg per ml for FG, and 2.53 ± 0.36 μg per ml for L3.6pl cells. Control Panc-1 cells was not affected by H-Zt/g4-MMAE with estimated IC_50_ > 80 μg/ml. The effect of H-Zt/g4-MMAE on PDAC cell death was shown in Fig. 2d. The IC_50_ of H-Zt/g4-MMAE in causing PDAC cell death ranged from 3.35 ± 0.26 μg per ml for BxPC-3, 2.71 ± 0.32 μg per ml for FG, and 1.37 ± 0.20 μg per ml for L3.6pl cells, respectively. These results demonstrate that H-Zt/g4-MMAE in vitro is highly effective in induction of RON internalization, which results in cell viability reduction followed by massive cell death.

### Pharmacokinetic profile of H-Zt/g4-MMAE in both mouse and cynomolgus monkey

**Fig. 3:**
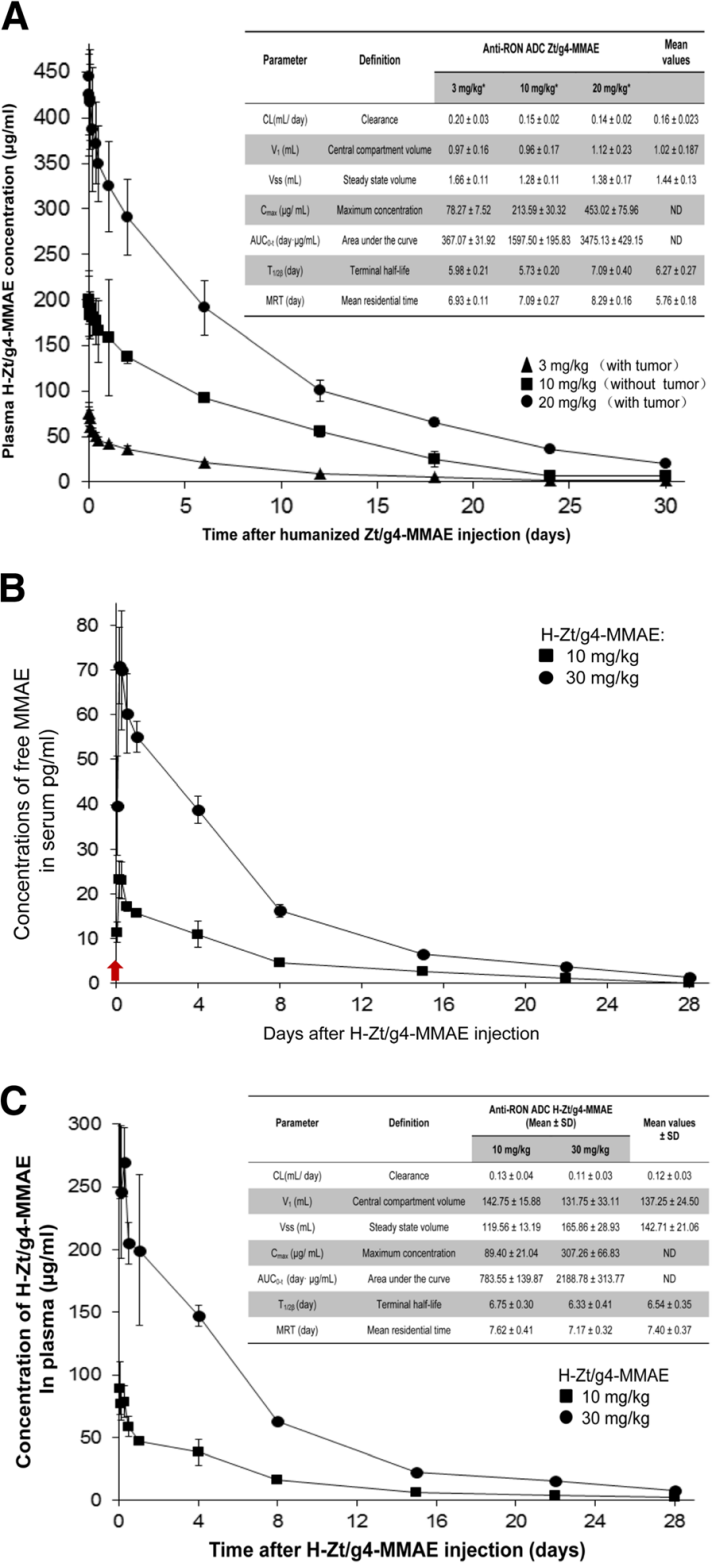
Pharmacokinetic profiles of H-Zt/g4-MMAE in both mouse and cynomolgus monkey: (**a**) PK profiles of H-Zt/g4-MMAE in mouse. Tumor-bearing and -nonbearing mice (athymic nude, 5 mice per group) were injected with a single dose of H-Zt/g4-MMAE at 3, 10, and 20 mg/kg, respectively. Collected blood samples were analyzed using the MMAE ADC ELISA kit (Eagle Biosciences, Inc., Nashua, NH). Various PK parameters were calculated using the software provided by Eagle Biosciences. (**b**) Free MMAE dissociated from H-Zt/g4-MMAE in monkey plasma. A single dose of H-Zt/g4-MMAE at 10 or 30 mg/kg was injected into cynomolgus monkey (3 animals per group). Free MMAE from individual blood samples collected at different time intervals were subjected to the LC-MS/MS analysis. (**c**) PK profiles of H-Zt/g4-MMAE in cynomolgus monkey. Blood samples from (**b**) were analyzed for MMAE coupled H-Zt/g4 using the MMAE ADC ELISA kit as described in (**a**) to obtain various PK parameters

The PKs of H-Zt/g4-MMAE in three doses were studied first in tumor-bearing and nonbearing mice to determine the time-dose relationship. Since H-Zt/g4 does not recognize mouse RON, the objectives were to determine: a) any alterations of the H-Zt/g4-MMAE PK profile in tumor-bearing mice and b) RON-independent behavior of H-Zt/g4-MMAE in tumor-nonbearing mice. Results in Fig. 3a show H-Zt/g4-NMAE in plasma in a two-compartment model from both tumor-bearing mice with a single dose of 3 and 20 mg/kg H-Zt/g4-MMAE and tumor-nonbearing mice with a single dose of 10 mg/kg H-Zt/g4-MMAE. Overall, the data from tumor-bearing mice were overlapped with those from the tumor-nonbearing mice with 95% prediction intervals. Thus, the PK of H-Zt/g4-MMAE is in no difference between tumor-bearing and nonbearing mice, suggesting that tumor growth does not affect the dynamics of H-Zt/g4-MMAE. Moreover, RON expression by tumor cells has no impact on H-Zt/g4-MMAE disposition in vivo.

The disposition of H-Zt/g4-MMAE was studied in cynomolgus monkey since this model is clinically relevant due to H-Zt/g4 specific binding to monkey RON. H-Zt/g4-MMAE at 10 or 30 mg/kg in a single dose was injected into animals. The maximal levels of free MMAE detected by the LC-MS/MS method were peaked around 3 h after ADC injection (Fig. 3b). The calculated free MMAE was ~ 25 pg and ~ 70 pg per ml plasma from 10 mg/kg and 30 mg/kg H-Zt/g4-MMAE treated animals, respectively, which were equivalent to ~ 0.58‰ of total MMAE conjugated to H-Zt/g4 from both doses. This decomposition ratio was steadily maintained during the period of the study, which fits the first order equation. As expected, free MMAE was gradually reduced in a time-dependent manner associated with reduction of H-Zt/g4-MMAE in vivo, which reflects their in vivo metabolic processes. At day 28, the levels of free MMAE in plasma were below 3 pg per ml plasma. These results suggest that the steady decomposition ratio is maintained in the plasma of cynomolgus monkey.

MMAE-conjugated H-Zt/g4 in plasma of cynomolgus monkey was measured to obtain a PK profile (Fig. 3c). The concentration of H-Zt/g4-MMAE in plasma fitted the two-compartment model. H-Zt/g4-MMAE had an average mean plasma clearance of 0.12 ml/day/kg, a t½ of 6.54 days, and a mean residential time of 7.40 days. These values were similar to those found in the mouse study (Fig. 3a). Nevertheless, there are differences in PK parameters between mouse and monkey due to physiological differences between two species (Fig. 3a and Fig. 3c). In conclusion, H-Zt/g4-MMAE is stable in vivo and the PK of H-Zt/g4-MMAE fits the two-compartment model in both species. Moreover, H-Zt/g4-MMAE disposition is not affected by endogenous RON expression by various tissues in cynomolgus monkey or by tumors that expressing RON.

### Superiority of H-Zt/g4-MMAE in eradication of PDAC tumors in multiple xenograft models

**Fig. 4:**
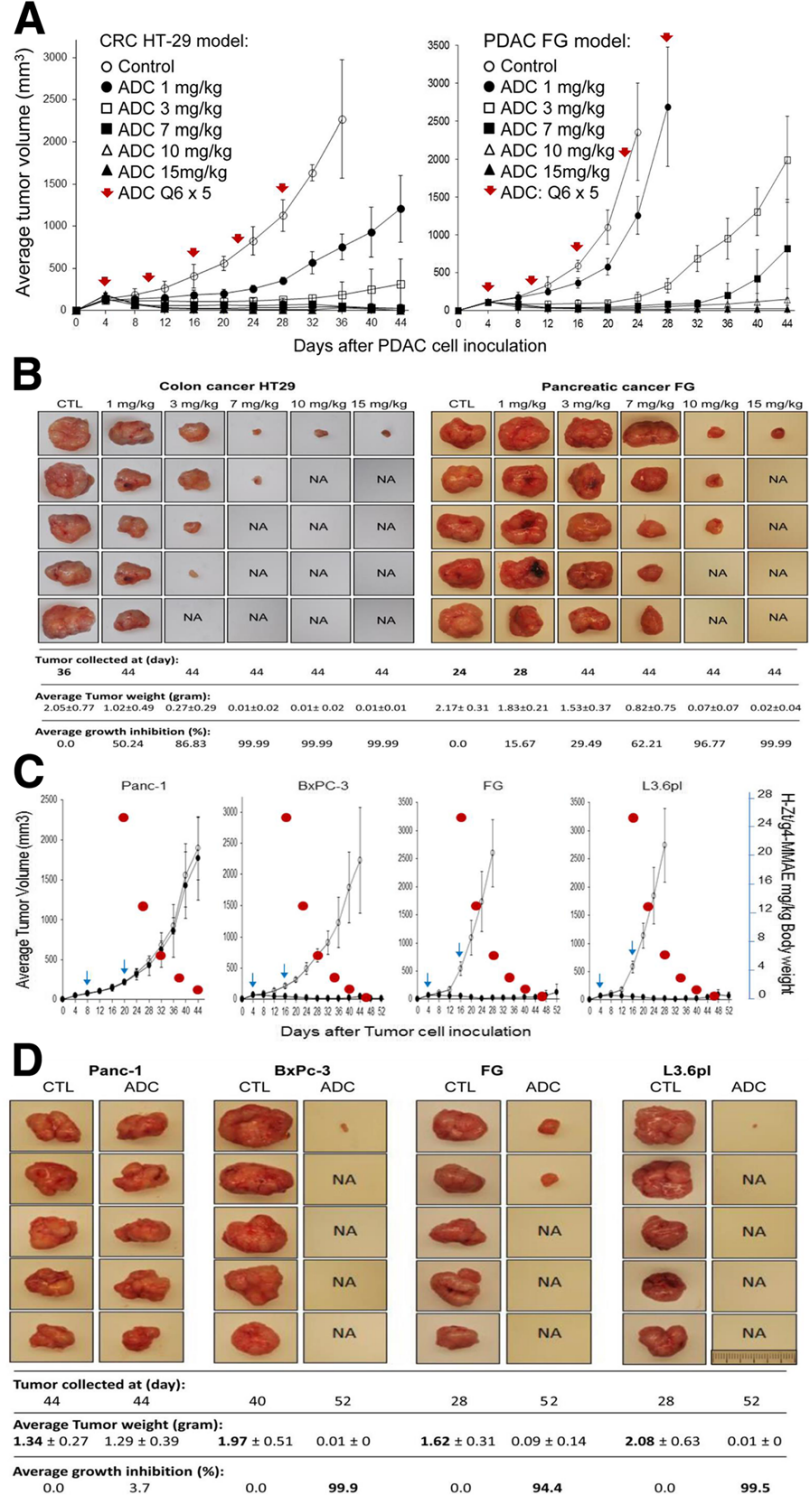
Therapeutic efficacy of H-Zt/g4-MMAE in PDAC xenograft tumor models: (**a**) Dose-dependent effect of H-Zt/g4-MMAE: Athymic nude mice (5 mice per group) were subcutaneously inoculated with 5 × 10^6^ FG cells. H-Zt/g4-MMAE at 1, 3, 7, 10, and 15 mg/kg was injected through tail vein in the Q6 × 5 regimen after tumors volumes reached to ~ 150 mm^3^. Mice injected with CmIgG-MMAE at 10 mg/kg were used as the control. Xenografts initiated by HT-29 cells served for comparison. (**b**) Effect of H-Zt/g4-MMAE in PDAC xenograft growth and eradication. Individual tumors from different groups described in (A) were collected from euthanized mice. Control mice bearing FG xenografts were sacrificed at day 24 due to rapid growth of tumors. Mice from other groups were killed at day 28 or day 44 dependent on the size of tumors. All tumors were weighted to reach the average tumor weight per group. The number of tumors from individual groups also was counted to determine the eradicating effect of H-Zt/g4-MMAE. NA, no tumors were found in the injected site. (**c**) Effect of H-Zt/g4-MMAE in three PDAC xenograft models: Xenograft tumors in mice (five animals per group) initiated by four PDAC cell lines were used for study. H-Zt/g4-MMAE was used at 20 mg/kg in the Q12 × 2 schedules. To establish the dose-effect relationship, the estimated reduction of H-Zt/g4-MMAE in vivo according to the t½ was marked as red circles. (**d**) Effect of H-Zt/g4-MMAE in tumor growth and eradication: Tumors were collected from mice described in (**b**). Tumor weight, count, and calculation were performed as described in (**b**). NA, no tumors were observed in the injected site

FG cell-mediated xenografts, which are fast growing and highly chemoresistant [44–46], were used to determine the dose-dependent effect of H-Zt/g4-MMAE. Xenografts caused by colorectal cancer (CRC) HT-29 cells served for comparison. The use of H-Zt/g4-MMAE at 1, 3, 7, 10, or 15 mg/kg in a Q6 × 5 schedule was based on ADC t½. H-Zt/g4-MMAE at 1 and 3 mg/kg is effective in delaying FG xenograft growth. The complete inhibition up to day 32 or 44 was found in mice receiving 7, 10, and 15 mg/kg H-Zt/g4-MMAE, respectively (Fig. 4a). Analysis of tumor number and weight showed that H-Zt/g4-MMAE not only inhibits tumor growth but also eradicates xenografts when it was used at high doses (Fig. 4b). Similar effects were also observed in HT-29 xenografts, which are more sensitive than FG models in response to H-Zt/g4-MMAE (Fig. 4a and b).

To verify the tumor-eradicating activity, H-Zt/g4-MMAE at 20 mg/kg in a Q12 × 2 schedule was used in xenograft models caused by three PDAC cell lines expressing variable levels of RON with different metastatic statuses [44–46]. Complete inhibition was observed during the course of the study (Fig. 4c). Analysis of tumor number and weight confirmed that H-Zt/g4-MMAE not only inhibits but also eradicates xenografts derived all three cell lines (Fig. 4d). The calculated tumor-static concentrations (TSC) for the residual xenografts derived from BxPc-3, FG, and L3.6pl cells were 0.5 mg/kg, 1.2 mg/kg, and 0.5 mg/kg of H-Zt/g4-MMAE, respectively. We noticed that levels of inhibition and/or eradication were comparable between chemoresistant L3.6pl and chemosensitive BxPC-3 xenograft models [44–46].

To determine if H-Zt/g4-MMAE is able to eliminate RON-expressing PDAC cells, Western blot analysis was performed to detect RON in tumor lysates from xenografts Levels of RON were progressively diminished in tumor lysates derived from FG cell-mediated tumors treated with H-Zt/g4-MMAE at different doses ((Additional file 1: Figure S2). Significantly, RON was not detected in tumors treated with 10 and 15 mg/kg H-Zt/g4-MMAE. These findings were further validated using tumor lysates from three PDAC xenografts (Fig. 4d), in which RON expression was barely detected in lysates from xenografts mediated by BxPC-3, FG, and L3.6pl cells after H-Zt/g4-MMAE treatment. Thus, H-Zt/g4-MMAE treatment results in elimination of RON expressing PDAC cells, indicating the target-specific action of ADCs.

We then tested H-Zt/g4-MMAE in inhibition of xenografts mediated by PDAC stem-like cells. CD24^+^/CD44^+^/epithelial-specific antigen (ESA)^+^ triple positive cells (PSC^+ 24/44/ESA^) possess many PDAC stem-like features with RON expression and were prepared from spheroid cells as previously described [38, 39]. We first determined the effect of H-Zt/g4-MMAE in vitro in killing PSC^+ 24/44/ESA^ (Additional file 1: Figure S3). The obtained IC_50_ values for PSC^+ 24/44/ESA^ were 1.78 μg/ml from BxPC-3 cells, 3.24 μg/ml from FG cells, and 2.93 μg/ml from L3.6pl cells, respectively, indicating H-Zt/g4-MMAE in vitro is effective in killing RON-positive PSC^+ 24/44/ESA^.

**Fig. 5:**
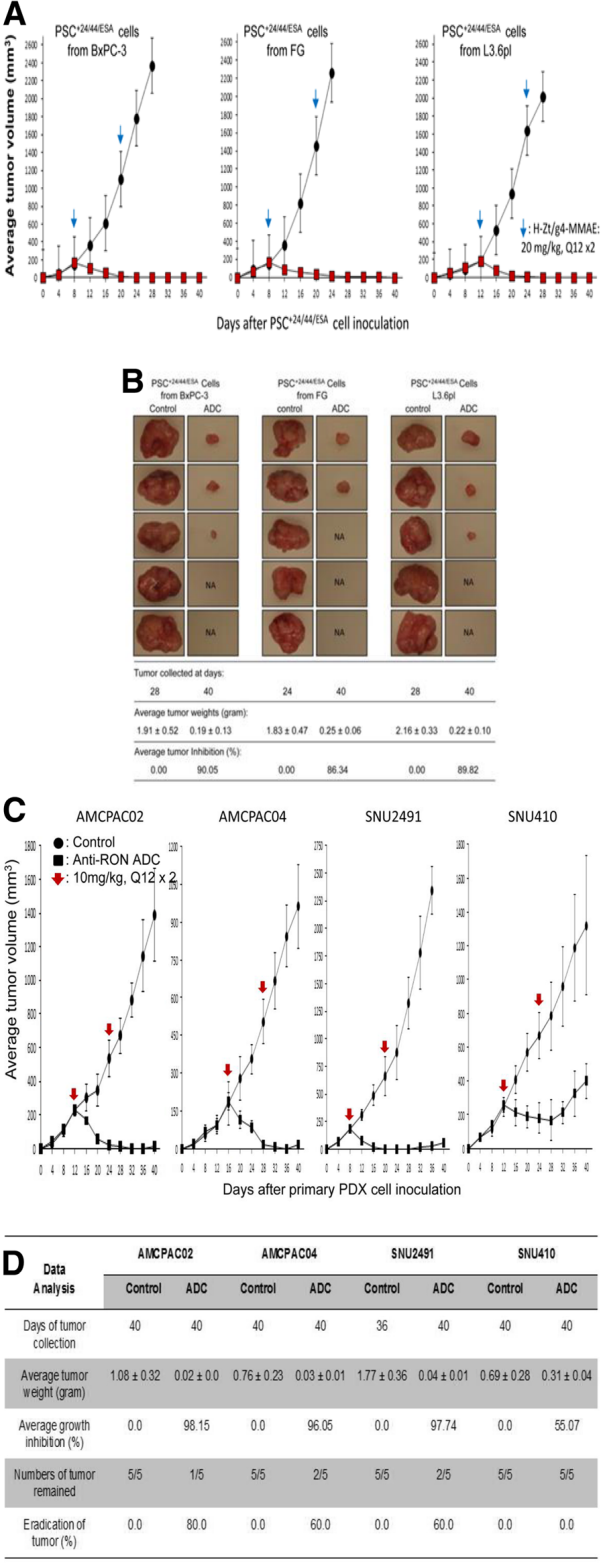
Therapeutic Effect of H-Zt/g4-MMAE on xenograft tumors mediated by PDAC stem-like cells and primary PDX cells: (**a**) Effect of H-Zt/g4-MMAE on PDAC stem-like cell derived xenografts: Athymic nude mice (five mice per group) were subcutaneously inoculated with 5 × 10^5^ PSC^+ 24/44/ESA^ prepared from BxPc-3, FG, and L3.6pl cells. H-Zt/g4-MMAE at 20 mg/kg was injected through tail vein in the Q12 × 2 regimen after tumors volumes reached to ~ 150 mm^3^. Mice injected with CmIgG-MMAE at 20 mg/kg were used as the control. (**b**) The eradicating effect of H-Zt/g4-MMAE on PDAC stem-like cell derived xenografts. Tumors were collected from mice as described in Fig. 4b. Tumor weight, count, and calculation were performed as described in Fig. 4b. (**c**) Mice were injected with individual primary PDX cell lines at 5 × 10^6^ cells in 0.1 ml in PBS. H-Zt/g4-MMAE at 10 mg/kg was injected through tail vein in the Q12 × 2 regimen after tumors volumes reached to 150 to 200 mm^3^. Mice injected with CmIgG-MMAE at 10 mg/kg were used as the control. (**d**) Individual tumors were collected from each group of mice as described in Fig. 4b. Average tumor weight and number per group were measured to determine levels of inhibition and eradication

Xenograft tumors were initiated by injection of 5 × 10^5^ PSC^+ 24/44/ESA^ into mice [39] followed by H-Zt/g4-MMAE treatment at 20 mg/kg in a 12 × 2 schedule. Complete inhibition with long-lasting effect up to 40 days was observed in PSC^+ 24/44/ESA^ xenografts derived from all three PDAC cell lines (Fig. 5a). Analysis of average tumor weights confirmed anti-PSC^+ 24/44/ESA^ xenograft activity of H-Zt/g4-MMAE (Fig. 5b) with weight reduction at 90.05, 86.34, and 89.82% for BxPC-3, FG, and L3.6pl PSC^+ 24/44/ESA^ models, respectively. Again, tumor eradication at variable levels was observed in PSC^+ 24/44/ESA^ xenografts (Fig. 5b). Western blot analysis confirmed diminished RON expression in residual cells from PSC^+ 24/44/ESA^ xenografts (Additional file 1: Figure S4).

The efficacy of H-Zt/g4-MMAE was further studied in PDAC patient-derived xenograft (PDX) models. Pathological and biological properties of eight PDX-derived primary PDAC cell lines [40] including their proliferation, RON expression and internalization, sensitivity to anti-RON ADC, and ability to grow in athymic nude mice are shown in Additional file 2: Table S1 and Additional file 1: Figure S5. H-Zt/g4-MMAE was effective in vitro in killing primary PDX cells expressing RON with IC_50_ values ranging from 1.75 to 10.31 μg/ml dependent on individual cell lines tested.

Four primary PDX cell lines including AMCPAC02, AMCPAC04, SNU2491, and SNU410 expressing variable levels of RON were used for in vivo study. Tumor growth from all four PDX cells was inhibited significantly following by H-Zt/g4-MMAE treatment at 10 mg/kg in a Q12 × 2 schedule (Fig. 5c and d). Collectively, TSCs were in the range of 1~3 mg/kg. By analyzing tumors at the end of study, we confirmed that H-Zt/g4-MMAE suppresses tumor growth with more than 95% of growth inhibition among three PXD models tested. Also, tumor eradication was documented (Fig. 5d). Xenografts from SNU410 cells were less sensitive to H-Zt/g4-MMAE with the TSC at 9.3 mg/kg, probably due to their low levels of RON expression (Additional file 2: Table S1 and Additional file 1: Figure S5A).

The efficacy of H-Zt/g4-MMAE was finally compared with H-Zt/g4-DM1 using PDAC and CRC xenograft as the model (Table 1**).** H-Zt/g4-DM1 was effective in inhibition of xenograft tumors mediated by three CRC cell lines; however, its effect on PDAC xenografts was moderate with significant inhibition seen only in non-metastatic BxPC-3 cells. Tumors from metastatic FG and L3.6pl cells were less sensitive to H-Zt/g4-DM1 (Table 1). In contrast, H-Zt/g4-MMAE exerted superior anti-tumor activity, which not only inhibits CRC cell-derived but also PDAC cell-derived xenograft tumors regardless their malignant status. The calculated inhibition with more than 98% was observed in all RON-positive xenografts. Thus, H-Zt/g4-MMAE is superior of H-Zt/g4-DM1 in inhibition of PDAC xenograft growth regardless tumor chemosensitivity and metastatic status.

**Table 1:** Therapeutic effect of H-Zt/g4-MMAE in comparison with H-Zt/g4-DM1 in inhibition of xenograft tumors derived from human pancreatic and colorectal cancer cells^a^

Anti-RON ADCs Evaluated	Tumor growth inhibition based on average tumor weights (gram)							
	Colorectal Cancer				Pancreatic cancer			
	LoVo	HCT116	HT29	SW620	Pan-1	BxPc-3	FG	L3.6pl
M-Zt/g4-DM1	ND	D16/D16:6.23/156 **(96.02%)**	D16/D16:5.92/180 **(96.67%)**	D16/D16:110/624 **(82.37%)**	ND	D32/D32: 0.06/0.7 **(91.8%)**	D20/D32: 1.89/2.49 **(24.1%)**	D16/D28: 1.41/2.54 **(44.6%)**
H-Zt/g4-DM1	D36/D36: 1.11/1.0 **(9.01%)**	D36/D36: 0.32/1.88 **(82.98%)**	D32/D36: 0.21/1.71 (**87.72%)**	D36/D36: 0.14/1.95 (**92.82%**)	D32/D32 1.25/1/32 **(−5.30%)**	D32/D32 0.08/0.81 (**90.12%)**	D32/d32 1.67/2.33 (**28.33%)**	D32/D32 1.31/2.44 (**46.31%)**
M-Zt/g4-MMAE	ND	ND	ND	ND	D44/44: 0.44/0,41 **(−7.32**)	D44/D52: 0.01/1.19 (**99.2%**)	D24/D44: 0.03/1.54 (**98.1**)	D24/D44: 0.02/1.58 (**98.7**)
H-Zt/g4-MMAE	D44/D44: 1.33/1.56 (**14.7%)**	D36/D52: 0.01/2.12 (**99.5%)**	D32/D52: 0.01/1.76 (**99.4%)**	D36/D52: 0.02/1.63 (**98.8%)**	D44/D44: 1.29/1.3 (**3.70%)**	D40/D52: 0.01/1.97 (**99.9%)**	D28/D52: 0.01/1.62 (**99.4%)**	D28/D52: 0.01/2.0 (**99.5%)**

^a^Anti-RON ADCs including mouse (M)-Zt/g4-DM1, H-Zt/g4-DM1, M-Zt/g4-MMAE, and H-Zt/g4-MMAE were prepared according to methods detailed in a previous report [31] and in Experimental Procedures. The drug to antibody ratios for each ADC are 3.72:1 for M-Zt/g4-DM1, 3.83:1 for H-Zt/g4-DM1, 3.67:1 for M-Zt/g4-MMAE, and 3.77:1 for H-Zt/g4-MMAE, respectively. CRC and PDAC xenografts in mouse model were established as detailed in Experimental Procedures. Individual ADCs were used at 20 mg/kg in the Q12 × 2 schedules. At the end of the study dependent on growth rate of individual models, tumors were collected and weighted to reach an average value for each group. The percentages of inhibition for tumor growth were calculated as detailed in Experimental Procedures

The percentages of inhibition were highlighted in bold

### Manageable toxicological activities of H-Zt/g4-MMAE in both mouse and cynomolgus monkey

Toxicological studies were performed first to determine the maximum tolerated dose in mouse by a single injection of H-Zt/g4-MMAE at 40, 60, 80, and 100 mg/kg. Mice receiving H-Zt/g4-MMAE up to 60 mg/kg showed normal daily activity, food consumption, and maintained bodyweight (Additional file 1: Figure S6). However, H-Zt/g4-MMAE at 80 mg/kg resulted in dramatic reduction of bodyweight. Furthermore, animal death (three out of five mice) occurred in animals receiving 100 mg/kg H-Zt/g4-MMAE. These studies imply that H-Zt/g4-MMAE up to 60 mg/kg is relatively safe in mice as judged by activity, food consumption, and bodyweight.

Cynomolgus monkey was then used to determine adverse activities by a single injection of H-Zt/g4-MMAE at 10 and 30 mg/kg, respectively. All cynomolgus monkeys survived after a single H-Zt/g4-MMAE injection at 10 mg/kg and 30 mg/kg. Abnormal changes were not observed grossly for daily activity, bodyweight, body temperature, food consumption, vision, electrocardiogram, breath, and urine samples. Histological analysis of multiple tissue/organs from individual monkeys did not find evidences of inflammation, hemorrhage, cell death, tissue damage, and weight reduction (Additional file 1: Figure S7).

**Fig. 6:**
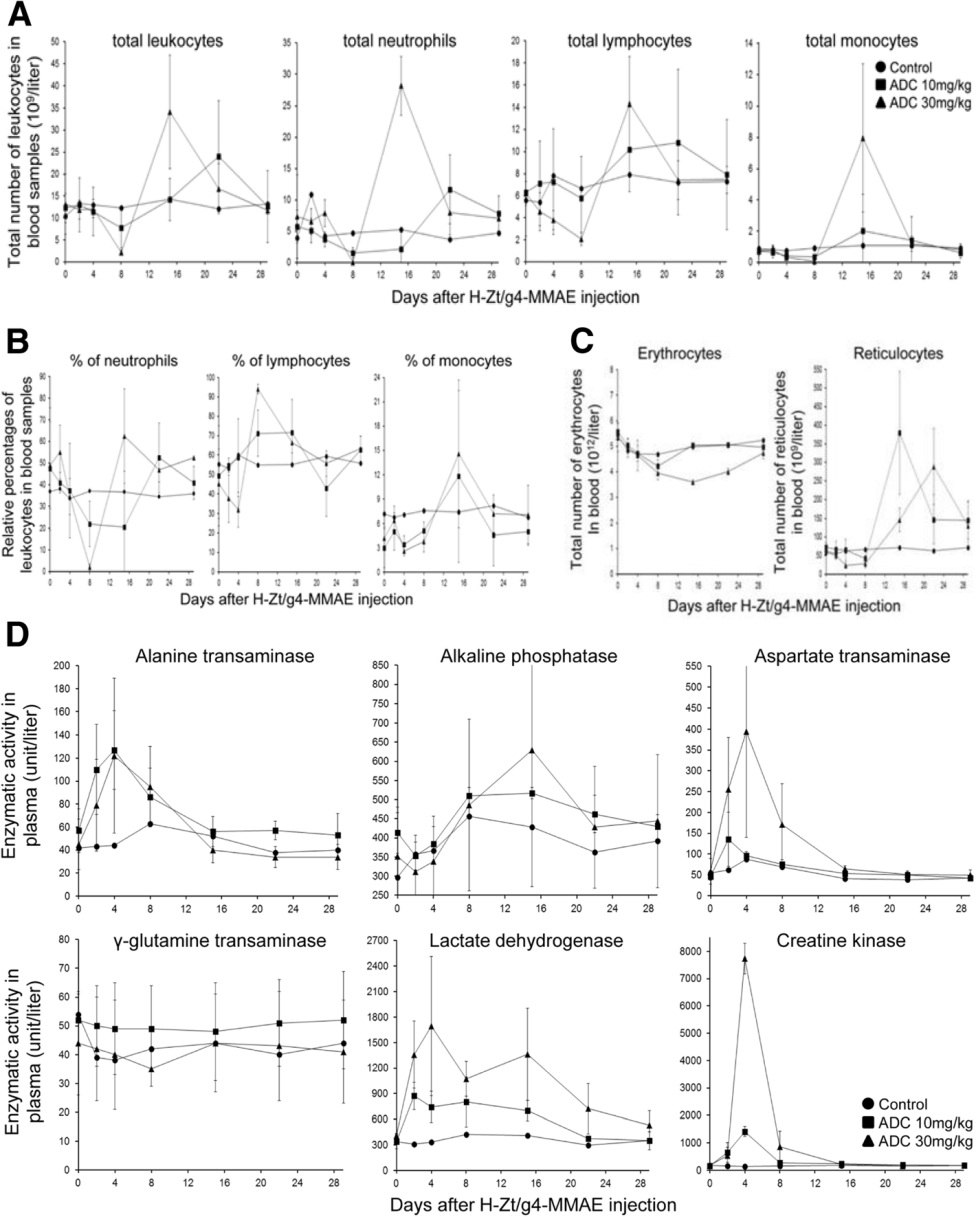
Toxicological activities of H-Zt/g4-MMAE in mouse and cynomolgus monkey. (**a**) and (**b**) Adverse activities of H-Zt/g4-MMAE in blood leukocytes in cynomolgus monkey. H-Zt/g4-MMAE at 10 or 30 mg/kg in a single dose was injected once into cynomolgus monkey. Monkeys without ADC injection served as the control. Peripheral blood samples were collected at different time intervals. Total numbers of leukocytes (**a**) including neutrophil, lymphocytes, and monocytes from each group were countered accordingly. The percentages of blood leukocytes (**b**) were also determined. (**c**) Adverse effect of H-Zt/g4-MMAE on blood erythrocytes and reticulocytes in cynomolgus monkey. Total numbers of erythrocytes and reticulocytes from blood samples collected from each group as described in (**b**) were counted accordingly. (**d**) Adverse effects of H-Zt/g4-MMAE on various enzymes in plasma of cynomolgus monkey. Six enzymatic activities from each group were quantitatively measured using the blood samples collected from individual monkeys as described in (**b**)

The adverse activities of H-Zt/g4-MMAE were documented from analysis of blood leukocytes, erythrocytes, reticulocytes (Fig. 6a, b, c, Additional file 2: Table S2), and liver function (Fig. 6d, and Additional file 2: Table S2). Leukocyte changes were featured by temporary and reversible reduction of the total number of leukocytes (Fig. 6a). H-Zt/g4-MMAE at 10 mg/kg at day 8 moderately decreased the total number of leukocytes (36.8%). Significant reduction (83.2%) was seen only when H-Zt/g4-MMAE reaches 30 mg/kg. Among three types of leukocytes analyzed, neutrophils were the most sensitive followed by monocytes and then lymphocytes (Fig. 6a and b). Due to the reduction of neutrophils, an increase in relative percentages of lymphocytes and monocytes was observed (Fig. 6a and b). It is worthy to note that the effect of H-Zt/g4-MMAE is nonspecific because blood leukocytes do not express RON [47]. The total number of leukocytes, including all three subtypes of leukocytes, was recovered to the normal level within ~ 20 days even H-Zt/g4-MMAE was used at 30 mg/kg. These results suggest that the hematopoietic system was not severely inhibited and/or impaired by H-Zt/g4-MMAE. Histological analysis of bone marrows from ADC-treated animals supported this notion (Additional file 1: Figure S7).

The H-Zt/g4-MMAE effect on blood erythrocytes was featured by reduction of total number of erythrocytes, which is accompanied with an increase in a delayed fashion in the total number of reticulocytes (Fig. 6c and Additional file 2: Table S2). The reduction in erythrocytes is ADC dose-dependent. H-Zt/g4-MMAE at 10 mg/kg slightly reduced the total number of erythrocytes, which was recovered to the normal level within 8 days (Fig. 6c). However, H-Zt/g4-MMAE at 30 mg/kg had a dramatic impact on the total number of erythrocytes (Fig. 6c), which resulted in an increase in the number of reticulocytes. In both cases, the effect on erythrocyte is reversible. The number of erythrocytes and reticulocytes recovered to the normal levels at the end of the study.

The adverse activity of H-Zt/g4-MMAE in liver function was evident by elevation of ALT, ALP, and AST (Fig. 6d and Additional file 2: Table S3). At day 29, levels of all enzymes were restored to the normal range. Hepatic histology examination did not find any evidence of liver structural damages (Additional file 1: Figure S7). A significant increase in LDH and CPK was observed in a dose-dependent manner after H-Zt/g4-MMAE injection (Fig. 5e), suggesting that functions of other tissues such as muscle could be impaired by H-Zt/g4-MMAE. Nevertheless, the elevated LDH and CPK levels were restored to the baseline within a short period, indicating the effect of H-Zt/g4-MMAE is temporary and reversible. Again, histological analysis of muscle and heart showing no pathological evidences of tissue damage (Additional file 1: Figure S7).

## Discussion

This report validates H-Zt/g4-MMAE as a lead candidate for targeted PDAC therapy. First, we developed H-Zt/g4-MMAE with a favorable pharmacological and PK properties in both mouse and monkey models. Second, we confirmed the efficacy in vivo showing that H-Zt/g4-MMAE at therapeutic doses in a single treatment cycle is sufficient to inhibit and eradicate xenograft tumors derived by established PDAC cells, PDAC stem-like cells, and primary cells from patient-derived tumors. Third, we profiled toxicological activities of H-Zt/g4-MMAE in both mouse and monkey models. The toxic activities were mainly restricted to hematopoietic system and liver, which are moderate, manageable and reversible. We conclude from these findings that H-Zt/g4-MMAE is superior with tumor-eradicating activity, which warrants for clinical trials in the future.

The use of mouse and cynomolgus monkey models for the PK profiling provides insight into the dynamics of H-Zt/g4-MMAE in vivo*.* We showed that the PK profile of H-Zt/g4-MMAE fits into the two-compartment model with the t½ of ~ 6.5 day in both animals, similar to other clinically approved ADCs such as T-DM1 [48, 49]. We found no differences in the dynamics of H-Zt/g4-MMAE between tumor-bearing and -nonbearing mice, indicating that tumor growth does not alter the H-Zt/g4-MMAE PK behavior [48, 49]. We further discovered that RON overexpression in xenograft tumors plays no role in impacting the fate of H-Zt/g4-MMAE in vivo. In addition, we demonstrated in cynomolgus monkey that the PK profiles of H-Zt/g4-MMAE are not affected by tissues/organs expressing RON. In other words, epithelial tissues constitutively expressing low levels of RON have very little impact on absorption, distribution, metabolism, and excretion of H-Zt/g4-MMAE. Taken together, these observations indicate that H-Zt/g4-MMAE has the favorable PK profile, which provides the pharmaceutical basis for use of H-Zt/g4-MMAE in clinical trials to determine its therapeutic efficacy.

The efficacy of H-Zt/g4-MMAE in vivo was confirmed using three PDAC xenograft models with different treatment regimens (Figs. 5 and 6). In xenografts mediated by FG cells, H-Zt/g4-MMAE at 1 mg/kg is able to delay tumor growth although its effect is relatively weak. Significant inhibition was observed only when ADC was used at 3 mg/kg. Interestingly, tumor eradication was observed when H-Zt/g4-MMAE was used at 10 and 15 mg/kg. These findings prompted us to apply H-Zt/g4-MMAE at 20 mg/kg in the Q12 × 2 schedule to maximize its therapeutic efficacy. Indeed, significant tumor eradication were observed in xenografts mediated by three PDAC cell lines after H-Zt/g4-MMAE treatment, highlighting the importance of using the relatively high doses of H-Zt/g4-MMAE in the initial phase to inhibit and to eradicate PDAC xenografts.

In xenografts mediated by PSC^+ 24/44/ESA^, we showed that H-Zt/g4-MMAE at 20 mg/kg in a Q12 × 2 regimen is sufficient to inhibit tumor growth mediated by PSC^+ 24/44/ESA^ derived from BxPC-3, FG, and L3.6pl cells. Moreover, tumor eradication was documented in this model. The finding from this study is important, which implies that H-Zt/g4-MMAE not only kills regular PDAC cells but also targets PDAC stem-like cells. The elimination of PDAC stem-like cells could provide therapeutic advantages in clinical setting.

Results from the third model of xenografts mediated by four primary PDX cell lines, confirmed that H-Zt/g4-MMAE is superior in PDAC inhibition and/or eradication. The use of primary cells from PDX is attractive because xenografts generated from these cells usually maintain their original molecular signature and malignant phenotype [40]. We showed that primary PDAC cells from PDXs are the target of H-Zt/g4-MMAE. The patterns of tumor inhibition and/or eradication are similar to those observed in the first and second models, indicating that H-Zt/g4-MMAE targeting RON for PDAC therapy has clinical relevance.

Analysis of toxicological profiles in cynomolgus monkey helps to identify tissues and/or organs affected by H-Zt/g4-MMAE. We used two doses in a single injection to determine H-Zt/g4-MMAE toxicity. Analyses by monitoring daily activity, bodyweight, body temperature, food consumption, heart rate, breath, vision, and urination did not find evidence-based abnormalities. Also, analysis of electrocardiogram, urine sample, and histology of tissues at the end-point find no evidence of tissue inflammation, cell death, structural alteration, hemorrhage, and other pathological changes in all animals tested. Nevertheless, changes were observed in blood chemistry tests showing adverse activities affecting hematopoietic system and liver function. The adverse effects were evident by moderate increase and/or decrease in blood leukocytes, reticulocytes, and a panel of liver enzymatic activities in H-Zt/g4-MMAE treated monkeys, which are dose-dependent, reversible, and manageable. At the end of the study, all changes were restored to the baseline. Due to the short length of studies, the toxic effect of H-Zt/g4-MMAE on animal reproductive tissues was not examined. In reviewing literatures related to ADC toxicity, we notice that toxic profiles of H-Zt/g4-MMAE are highly similar to those of ADCs approved by FDA or currently under clinical trials [50]. Specifically, toxicities of ADCs conjugated with tubulin inhibitors such as MMAE all have a similar profiles affecting the hematopoietic system, liver, and reproductive organs regardless their reactivity to target antigens [50]. For examples, among five ADCs conjugated with MMAE tested in cynomolgus monkeys analyzed by FDA, the prominent organ toxicities are observed mainly in hematopoietic system, liver, and reproductive organs [50]. Considering these facts, we reason that H-Zt/g4-MMAE generated through classical drug-linkage technology is relatively safe when used in therapeutic doses. Furthermore, the preclinical data presented in this study should help to design a phase 1 clinical trials for H-Zt/g4-MMAE.

## Additional files

Additional file 1: **Figure S1.** Immune reactivity of H-Zt/g4 to RON in multiple tissues from human and cynomolgus monkey. **Figure S2.** Effect of H-Zt/g4-MMAE on eradication of RON-expressing cells in PDAC xenograft tumors. **Figure S3.** Induction in vitro by H-Zt/g4-MMAE of death of PDAC stem-like cells. **Figure S4**. Effect of H-Zt/g4-MMAE on elimination of RON-expressing cells in PSC + 24/44/ESA cell-mediated xenografts. **Figure S5A.** Cell surface RON expression by a panel of primary PDX cell lines. **Figure S5B.** Induction of cell surface RON internalization by H-Zt/g4. **Figure S5C.** Effect of H-Zt/g4-MMAE on viability of primary PDAC cell lines derived from patient’ tumors. **Figure S6.** Histological examination of multiple organs and/or tissues from cynomolgus monkey treated with H-Zt/g4-MMAE. **Figure S7.** Histological examination of multiple organs and/or tissues from cynomolgus monkey treated with H-Zt/g4-MMAE. (PDF 2315 kb)

Additional file 2: **Tables S1.** Pathological and Biological Features of Primary PDAC Cell Lines from Patient-Derived Xenograft Tumors*. **Table S2.** Adverse Effects of H-Zt/g4-MMAE on blood leukocyte and erythrocytes in Cynomolgus monkey. **Table S3.** Effect of H-Zt/g4-MMAE in vivo on various enzymatic activities in blood samples collected from cynomolgus monkeys. (PDF 663 kb)

## Abbreviations

ADC: Antibody-drug conjugates
CDR: Complementarity-determining region
CRC: Colorectal cancer
DAPI: 4′,6-diamidino-2-phenylindole
DAR: Drug to antibody ratio
DM1: Maytansinoid derivative 1
ELISA: Enzyme-linked immunosorbent assay
ESA: Epithelial-specific antigen
FITC: Fluorescein isothiocyanate
HIC: Hydrophobic interaction chromatography
LAMP 1: Lysosomal-associated membrane protein 1
LC-MS/MS: Liquid Chromatography with tandem mass spectrometry
mAb: Monoclonal antibodies
MET: Mesenchymal-epithelial transition
MMAE: Monomethyl auristatin E
MMAF: Monomethyl auristatin F
MTT: 3-(4, 5- dimethylthiazolyl-2)-2, 5diphenyltetrazolium bromide
PDAC: Pancreatic ductal adenocarcinoma
PK: Pharmacokinetic
RON: Recepteur d’origine nantais
SMKI: Small-molecule kinase inhibitors
TNBC: Triple-negative breast cancer
