# Correction to: Therapeutic efficacy, pharmacokinetic profiles, and toxicological activities of humanized antibody-drug conjugate Zt/g4-MMAE targeting RON receptor tyrosine kinase for cancer therapy

**DOI:** 10.1186/s40425-019-0571-7

**Published:** 2019-04-02

**Authors:** Hang-Ping Yao, Liang Feng, Sreedhar Reddy Suthe, Ling-Hui Chen, Tian-Hao Weng, Chen-Yu Hu, Eun Sung Jun, Zhi-Gang Wu, Wei-Lin Wang, Song Cheol Kim, Xiang-Min Tong, Ming-Hai Wang

**Affiliations:** 1 State Key Laboratory for Diagnosis & Treatment of Infectious Diseases, First Affiliated Hospital, Zhejiang University School of Medicine, Hangzhou, China; 2 Cancer Biology Research Center, Hangzhou, China; 3 Department of Pharmaceutical Sciences, Texas Tech University Health Sciences Center School of Pharmacy, Amarillo, TX USA; 4 Zhejiang Provincial Key Laboratory for Precision Diagnosis & Treatment of Hepatic & Pancreatic Oncology, Hangzhou, Zhejiang Province China; 5 Division of Hepatobiliary & Pancreatic Surgery, First Affiliated Hospital, Zhejiang University School of Medicine, Hangzhou, China; 6 Department of Convergence Medicine, Asan Institute for Life Sciences, University of Ulsan College of Medicine and Asan Medical Center, Seoul, Republic of Korea; 7 Division of Hepato-Biliary and Pancreatic Surgery, Department of Surgery, University of Ulsan College of Medicine, Asan Medical Center, Seoul, Republic of Korea; 8 Department of Laboratory Medicine, Zhejiang Provincial People’s Hospital, Hangzhou Medical College, Hangzhou, China

**Correction to: J Immunother Cancer 2019 7:75**

**https://doi.org/10.1186/s40425-019-0525-0**

Following publication of the original article [1], the author reported two errors in the authors affiliations:

1. Dr. Song Cheol Kim should be affiliated to Division of Hepato-Biliary and Pancreatic Surgery, Department of Surgery,University of Ulsan College of Medicine, Asan Medical Center, Seoul, Republic of Korea;

2. Dr. Eun Sung Jun should be affiliated to Department of Convergence Medicine, Asan Institute for Life Sciences, University of Ulsan College of Medicine and Asan Medical Center, Seoul, Republic of Korea and to Division of Hepato-Biliary and Pancreatic Surgery, Department of Surgery,University of Ulsan College of Medicine, Asan Medical Center, Seoul, Republic of Korea;

## References

[1] Yao et al. (2019). Therapeutic efficacy, pharmacokinetic profiles, and toxicological activities of humanized antibody-drug conjugate Zt/g4-MMAE targeting RON receptor tyrosine kinase for cancer therapy. Journal for ImmunoTherapy of Cancer.

