# Supplementary material for: Therapeutic efficacy, pharmacokinetic profiles, and toxicological activities of humanized antibody-drug conjugate Zt/g4-MMAE targeting RON receptor tyrosine kinase for cancer therapy
Source: J Immunother Cancer. 2019 Mar 14;7:75. doi: 10.1186/s40425-019-0525-0 (PMC6419354; doi:10.1186/s40425-019-0525-0)
Supplement: Supplementary file 1 — Figure S1. Immune reactivity of H-Zt/g4 to RON in multiple tissues from human and cynomolgus monkey. Figure S2. Effect of H-Zt/g4-MMAE on eradication of RON-expressing cells in PDAC xenograft tumors. Figure S3. Induction in vitro by H-Zt/g4-MMAE of death of PDAC stem-like cells. Figure S4. Effect of H-Zt/g4-MMAE on elimination of RON-expressing cells in PSC + 24/44/ESA cell-mediated xenografts. Figure S5A. Cell surface RON expression by a panel of primary PDX cell lines. Figure S5B. Induction of cell surface RON internalization by H-Zt/g4. Figure S5C. Effect of H-Zt/g4-MMAE on viability of primary PDAC cell lines derived from patient’ tumors. Figure S6. Histological examination of multiple organs and/or tissues from cynomolgus monkey treated with H-Zt/g4-MMAE. Figure S7. Histological examination of multiple organs and/or tissues from cynomolgus monkey treated with H-Zt/g4-MMAE. (PDF 2315 kb) [file 40425_2019_525_MOESM1_ESM.pdf]

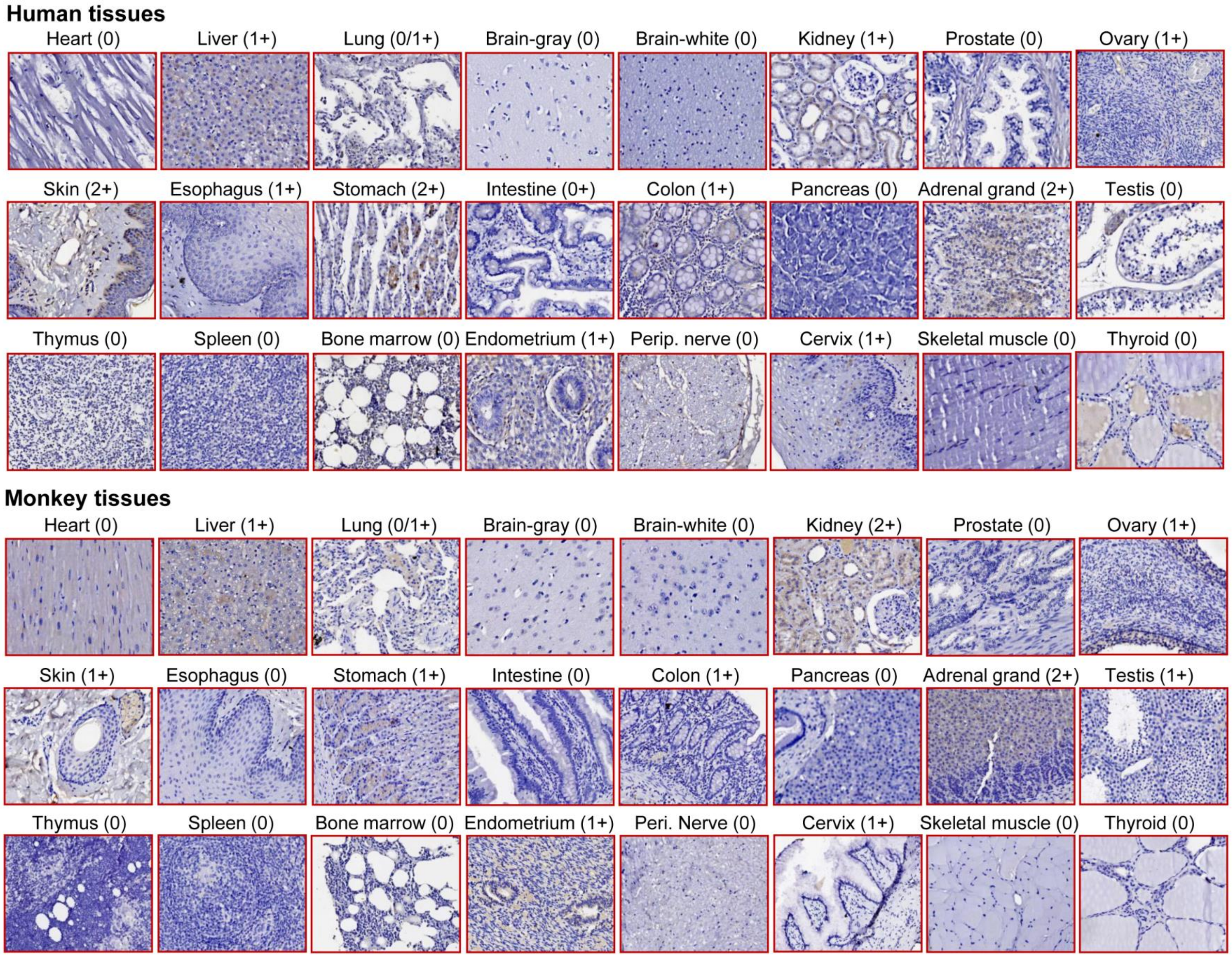

Yao et al: Supplementary Figure 1

Supplementary Fig. 1: Immune reactivity of H-Zt/g4 to RON in multiple tissues from human and cynomolgus monkey. Multiple normal tissues from human and cynomolgus monkey formatted in a high condensed tissue array were obtained from US Biomax, Inc (www.biomax.us). IHC staining was performed using H-Zt/g4 at 4 μg/ml followed by DAKO EnVision System as previously described [9]. Immunoreactivity of RON was determined by specific cytoplasmic and membrane staining. Levels of RON expression in different tissues were determined by the semi-quantitative IHC method as described previously [9,33]

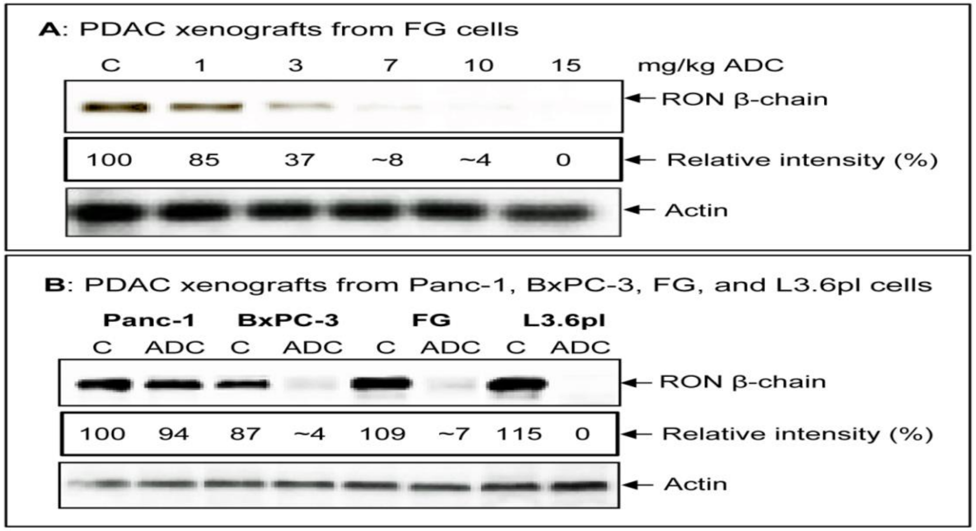

Supplementary Fig. 2: Effect of H-Zt/g4-MMAE on eradication of RON-expressing cells in PDAC xenograft tumors. **(A)** Cellular proteins (20 µg per sample) from tumor lysates of FG cell-derived xenograft tumors were separated in the 8% SDS-PAGE under reduced conditions. RON was detected by rabbit IgG antibody R#5029 specific to the RON C-terminal peptide. Levels of RON from individual samples were analyzed using densitometry. **(B)** Xenografts mediated by Panc-1, BxPC-3, FG, and L3.6pl cells were used for study. Tumor lysate preparation, Western blot analysis, and measurement of RON proteins were performed as described in (A).

Yao HP et al. Supplementary Figure 2

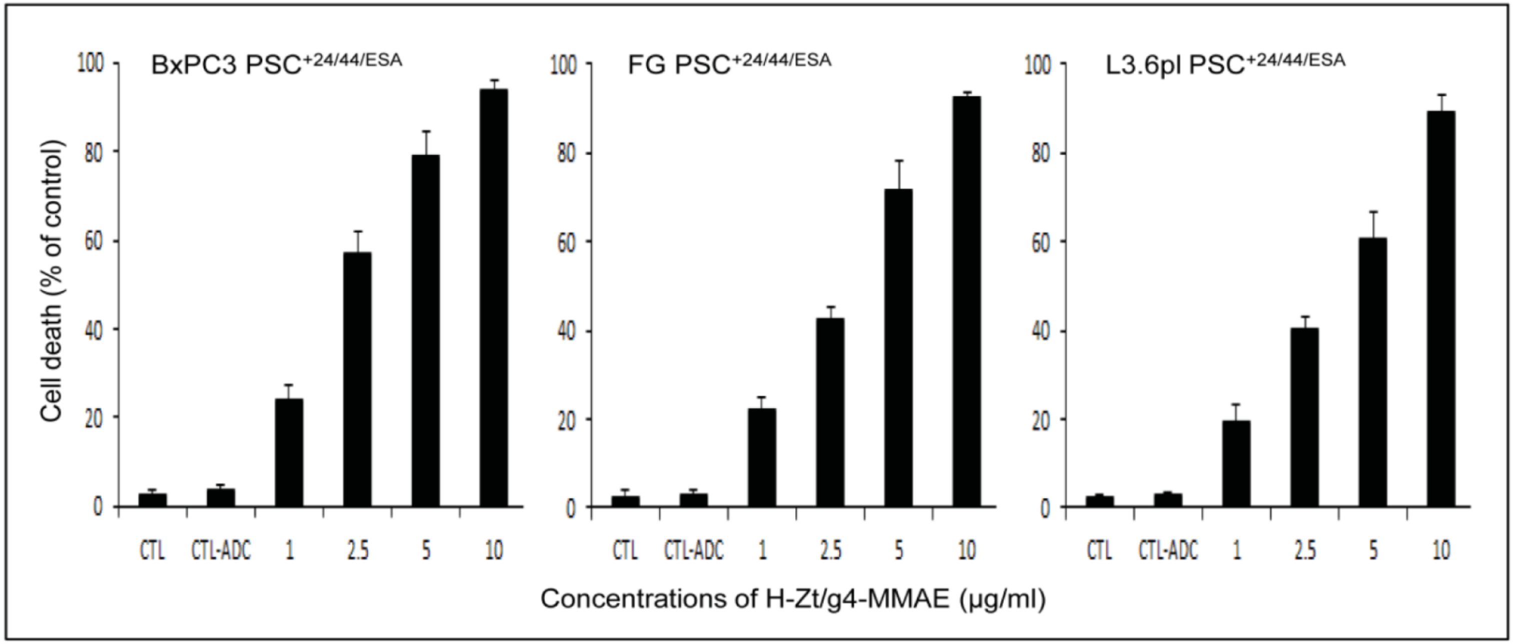

Supplementary Fig. 3 Induction in vitro by H-Zt/g4-MMAE of death of PDAC stem-like cells. PSC$^{+24/44/ESA}$ cells from BxPC-3, FG and L3.6pl cells (8000 cells per well in a 96-well plate in triplicate) were treated with different amounts of H-Zt/g4-MMAE for 96h. The percentages of cell death were determined by the trypan blue exclusion method. Data shown here are derived from one of two experiments with similar results.

Yao HP et al. Supplementary Fig. 3

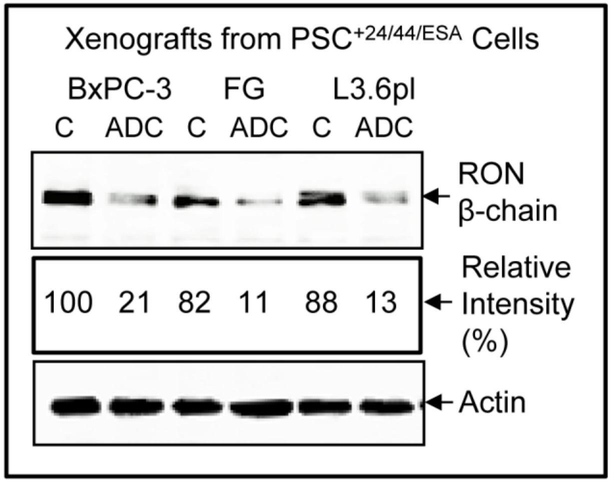

Supplementary Fig. 4: Effect of H-Zt/g4-MMAE on elimination of RON-expressing cells in PSC+24/44/ESA cell-mediated xenografts. Xenografts mediated by PSC$^{+24/44/ESA}$ cells prepared from BxPC-3, FG, and L3.6pl cells were used for study. Tumor lysate preparation, Western blot analysis, and measurement of RON proteins were performed as described in Supplementary Fig. 2.

Yao HP et al. Supplementary Figure 4

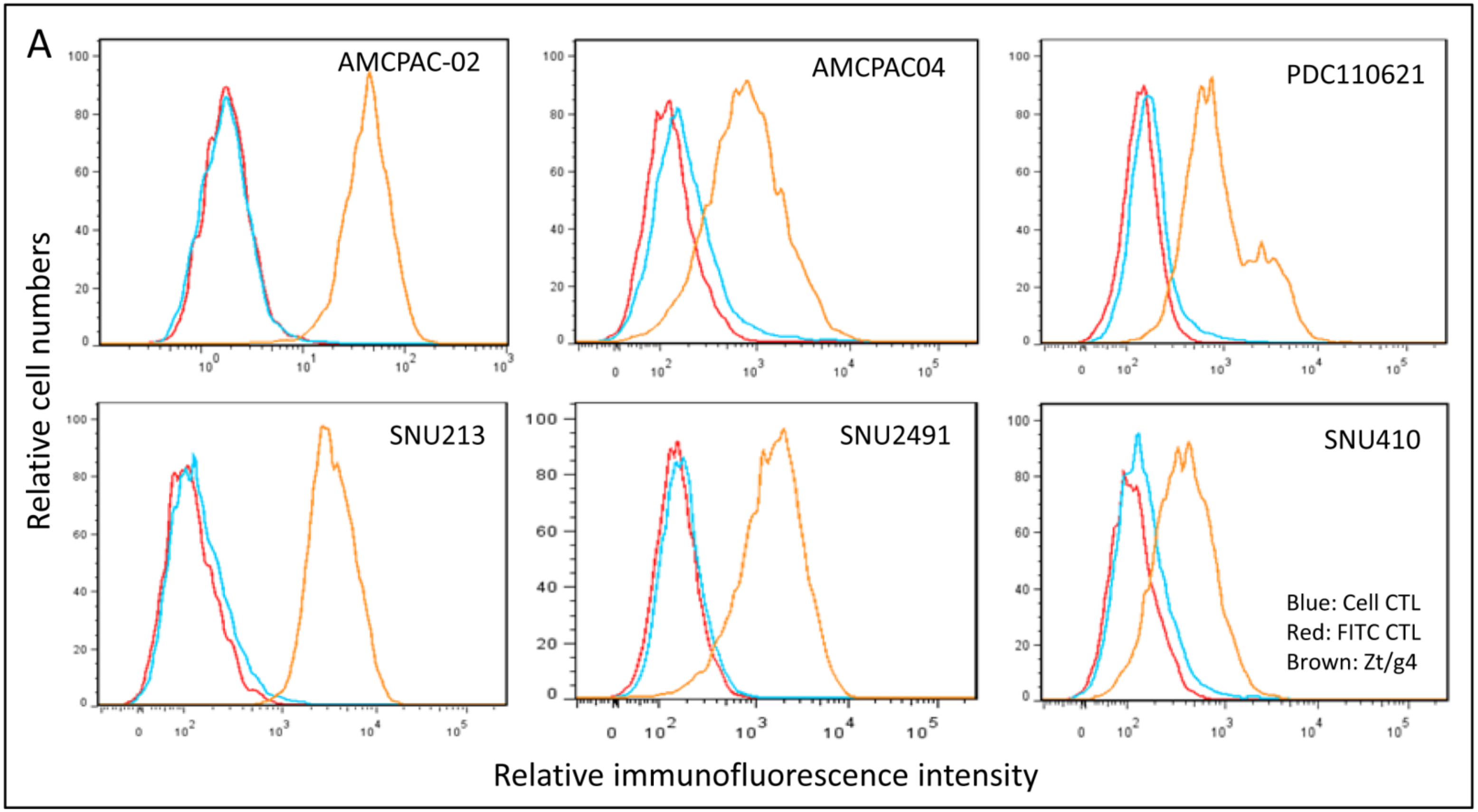

Supplementary Fig. 5A: Cell surface RON expression by a panel of primary PDX cell lines. A panel of primary cell lines established from PDX was used as previously described [49]. Cells at 1 x10^6 cells per ml in PBS were incubated with 2 µg/ml of H-Zt/g4 for 45 min at 4°C followed by addition of goat antihuman IgG1 coupled with FITC. Cells treated with isotype-matched human IgG1 or with goat anti-human IgG-coupled with FITC alone were used as the control. Fluorescence intensities from individual samples were determined by flow cytometric analysis as previously described [30].

Yao HP et al. Supplementary Figure 5A

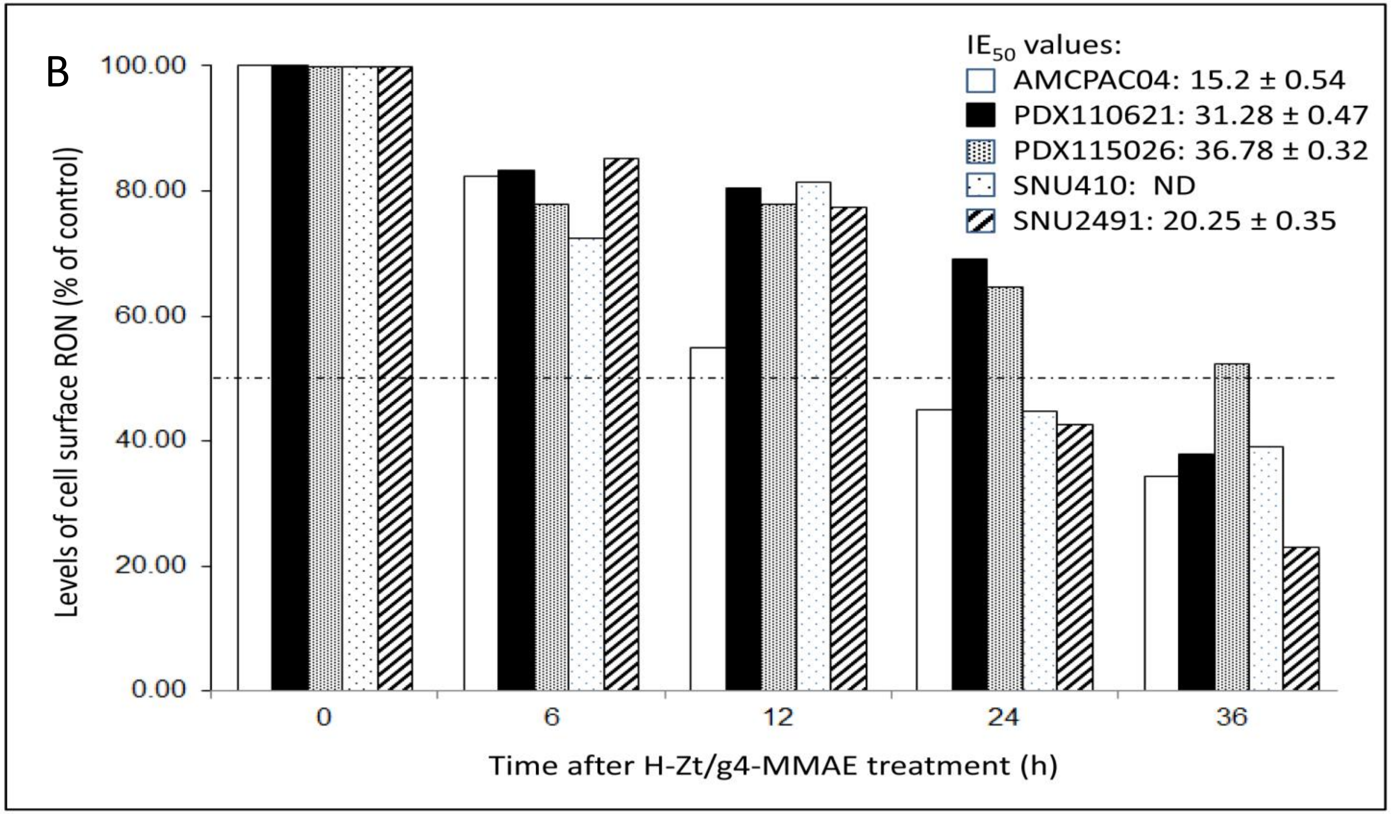

Supplementary Fig. 5B: Induction of cell surface RON internalization by H-Zt/g4. Primary PDX cell lines (1 x10^6 cells per dish) were treated at 37°C with 5 µg/ml of H-Zt/g4, collected at different time points, washed with acidic buffer to eliminate cell surface bound IgG, and then incubated with 2 µg/mL of anti-RON mAb Zt/c1 [34]. Immunofluorescence intensity was analyzed by flow cytometer using FITC-coupled goat anti-mouse IgG. Immunofluorescence intensities from cells treated with H-Zt/g4 at 4°C were set as 100%. Levels of RON remained on the cell surface was determined as previously described [31].

Yao HP et al. Supplementary Figure 5B

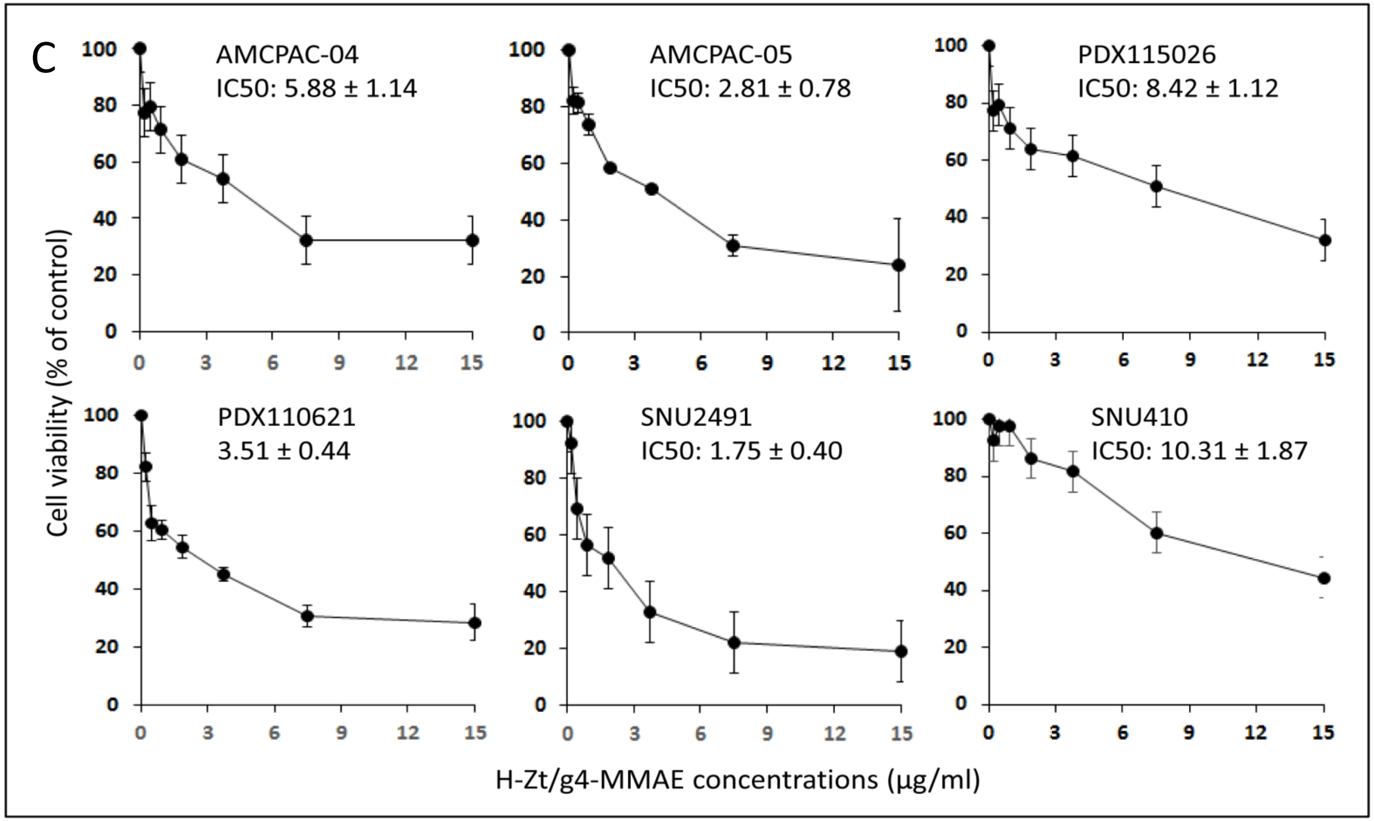

Supplementary Fig. 5C. Effect of H-Zt/g4-MMAE on viability of primary PDAC cell lines derived from patient' tumors. Six primary PDAC cell lines (8000 cells per well in a 96-well plate in triplicate) were treated with different amounts of H-Zt/g4-MMAE for 96h. Cell viability was determined by the MTS assay as previously described [31]. Results shown here are from one of three experiments with similar results.

Yao HP et al. Supplementary Figure 5C

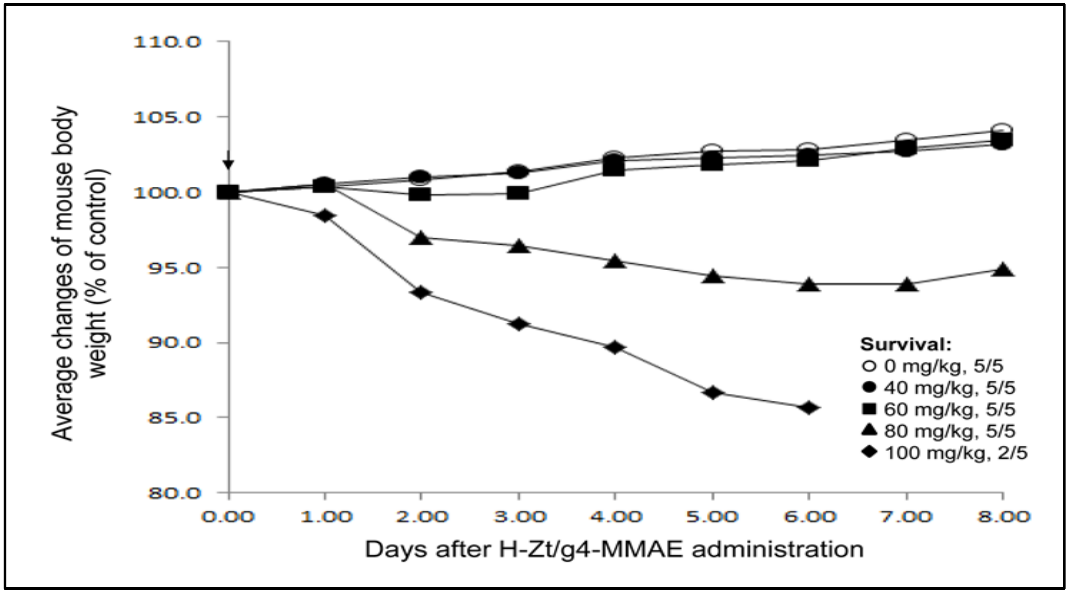

Supplementary Fig. 6 Effect of H-Zt/g4-MMAE in mouse body weight and death: Dose-dependent H-Zt/g4-MMAE toxicity in mouse. H-Zt/g4-MMAE at 40, 60, 80, and 100 mg/kg in a single dose was injected into mice (5 animals per group). Mice without ADC injection were used as the control. The body weight and survival were monitored every day up to eight days. The average body weight from each group before ADC injection was set as 100%. Percentages of the average body weight reduction were calculated as: 100% - (average body weight after ADC injection/average body weight before ADC injection x 100%).

Yao HP et al. Supplementary Fig. 6

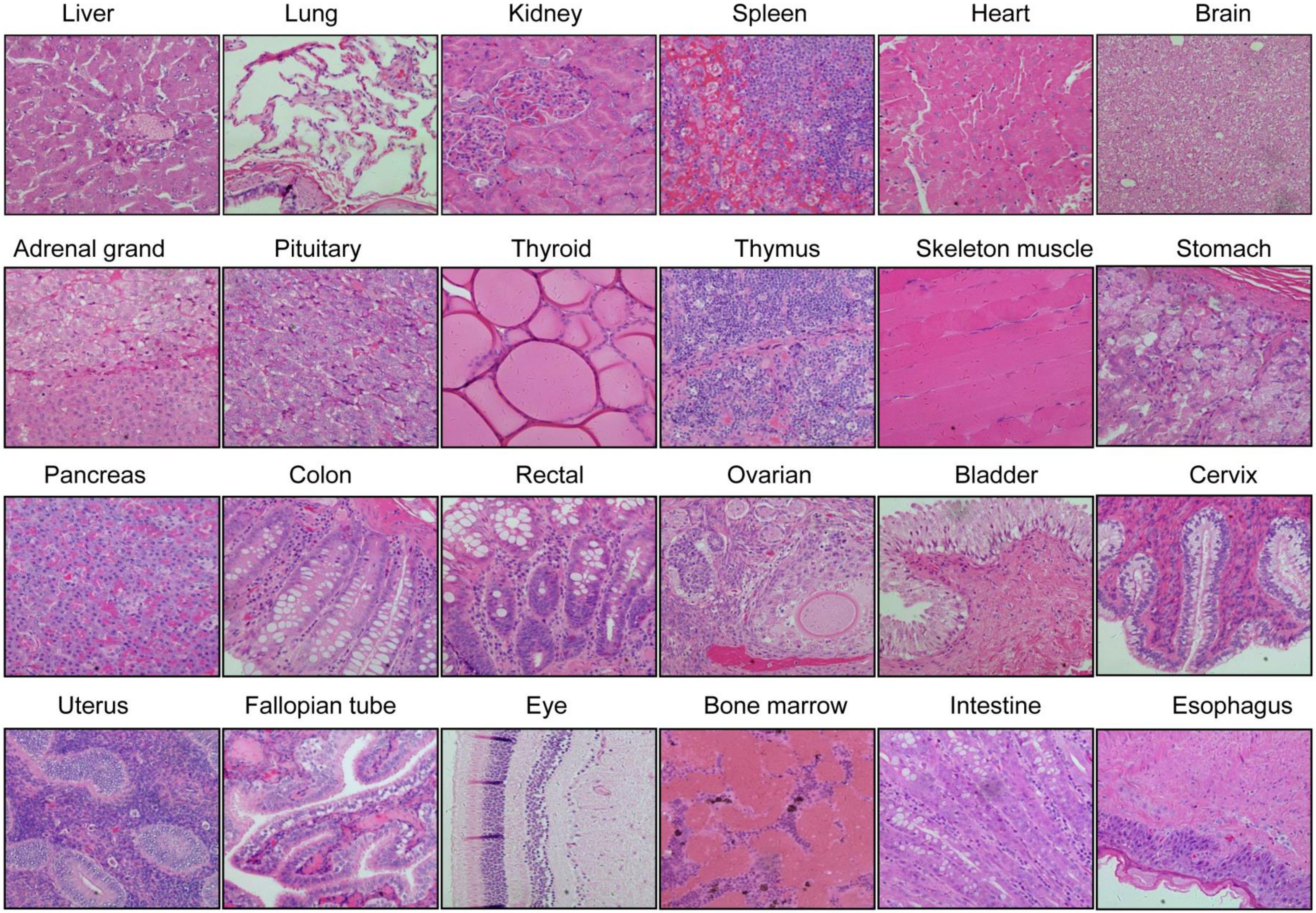

Yao HP et al. Supplementary Figure 6

Supplementary Fig. 6: Histological examination of multiple organs and/or tissues from cynomolgus monkey treated with H-Zt/g4-MMAE. Cynomolgus monkeys (three animals per group) were treated with a single injection of 10 or 30 mg/kg H-Zt/g4-MMAE through saphenous vein. Animals were monitored daily for 29 days and then sacrificed. Three animals without ADC served as the control. All monkeys were subjected to histological examination at the end of the study. Various organs and tissues were collected and processed for histological analysis. Tissue slides were evaluated by pathologists from Department of Pathology in the First Hospital affiliated with Zhejiang University School of Medicine (Hangzhou, China) for any evidence of inflammation, hemorrhage, cell death, tissue damage, structural alteration, size change, and weight reduction, which could be caused by H-Zt/g4-MMAE toxicity. Detailed observation did not find any pathological abnormalities in all tissues/organs from all monkeys. The representative images from one cynomolgus monkey injected with 30 mg/kg H-Zt/g4-MMAE was shown here. Similar results were also observed in control and experimental animals injected with 10 or 30 mg/kg ADC.

Yao HP et al. Supplementary Figure 6
