# Supplementary material for: Therapeutic efficacy, pharmacokinetic profiles, and toxicological activities of humanized antibody-drug conjugate Zt/g4-MMAE targeting RON receptor tyrosine kinase for cancer therapy
Source: J Immunother Cancer. 2019 Mar 14;7:75. doi: 10.1186/s40425-019-0525-0 (PMC6419354; doi:10.1186/s40425-019-0525-0)
Supplement: Supplementary file 2 — Tables S1. Pathological and Biological Features of Primary PDAC Cell Lines from Patient-Derived Xenograft Tumors*. Table S2. Adverse Effects of H-Zt/g4-MMAE on blood leukocyte and erythrocytes in Cynomolgus monkey. Table S3. Effect of H-Zt/g4-MMAE in vivo on various enzymatic activities in blood samples collected from cynomolgus monkeys. (PDF 663 kb) [file 40425_2019_525_MOESM2_ESM.pdf]

Supplementary Table 1 Pathological and Biological Features of Primary PDAC Cell Lines from Patient-Derived Xenograft Tumors*

PDAC Cell lines	Patient age & gender	Tumor size & stage	Lymphatic invasion	Morphology of cell lines	Doubling time (h)	RON expression	Endocytosis of RON (EC_{50} = h)	Sensitivity to ADC (µg/ml)	Growth in athymic mice
**AMC-01**	66 & M	3.5 & IIA	Yes	epithelial-like	68	++++	Not done	2.13 ± 0.25	Not observed
**AMC-02**	30 & M	2.1 & IIA	No	epithelial-like	87	++++	14.3 ± 0.76	1.98 ± 0.33	Able to grow
**AMC-04**	53 & M	3.1 & IIB	No	mesenchymal-like	48	++	15.2 ± 0.54	5.88 ± 1.14	Able to grow
**AMC-05**	70 & M	5.7 & IIA	Yes	epithelial-like	75	+++	ND	2.81 ± 0.78	Not done
**PDC110621**	66 & M	3.5 & IIA	Yes	epithelial-like	ND	++	31.28 ± 0.47	3.51 ± 0.44	Able to grow
**PDC115026**	70 & M	5.7 & IIB	Yes	epithelial-like	ND	+++	36.78 ± 0.32	8.42 ± 1.12	Not done
**SNU2491**	61 & F	2.5 & IIB	Yes	epithelial-like	64	+++	20.25 ± 0.35	1.75 ± 0.40	Able to grow
**SNU410**	53 & M	NA &V	No	mesenchymal-like	72	+	Not observed	10.31 ± 1.87	Able to grow

*Primary cell lines from PDXs of PDAC patients were generated and characterized as detailed in a previous report (Jung et al., 2016). Levels of RON expression by individual cell lines were determined using immunofluorescence and Western blot analyses. H-Zt/g4 was used as the primary antibody. H-Zt/g4-induced RON endocytosis was performed using the method detailed in Experimental Procedures. Sensitivity of individual cell lines to H-Zt/g4-MMAE-induced reduction in cell viability was performed using the MTS assay as described previously [31]. Individual cell lines having capable of growing in athymic nude mice were selected for *in vivo* H-Zt/g4-MMAE therapeutic study.

Supplementary Table 2 Adverse Effects of H-Zt/g4-MMAE on blood leukocyte and erythrocytes in Cynomolgus monkey

Measurement of blood cells	Treatment with ADC	Dynamic changes of leukocytes and erythrocytes after H-Zt/g4-MMAE injection (days, Mean ± SD)						
		0	2	4	8	15	22	29
Total leukocytes (10^9/liter)	Control	10.37 ±	13.35 ±	12.94 ±	12.35 ±	14.33 ±	12.12 ±	13.15 ±
	10 mg/kg	12.29 ± 3.30	12.98 ± 6.16	11.47 ± 5.48	7.77 ± 4.64	14.23 ± 4.80	24.00 ± 12.63	12.62 ± 8.21
	30 mg/kg	13.15 ± 6.82	11.90 ± 2.18	12.11 ± 2.05	2.21 ± 0.57	34.11 ± 12.90	16.68 ± 5.68	11.71 ± 0.85
Total neutrophils (10^9/liter)	Control	3.93 ±	10.88 ±	4.27 ±	4.70 ±	5.24 ±	3.71 ±	4.68 ±
	10 mg/kg	5.77 ± 2.64	5.12 ± 1.97	3.70 ± 1.14	1.50 ± 0.86	2.18 ± 2.69	11.70 ± 5.53	7.85 ± 2.81
	30 mg/kg	7.34 ± 6.70	6.58 ± 2.07	7.89 ± 2.14	0.04 ± 0.03	28.18 ± 4.65	8.03 ± 3.63	7.08 ± 0.13
Total lymphocytes (10^9/liter)	Control	5.60 ±	5.43 ±	7.83 ±	6.67 ±	7.90 ±	7.18 ±	7.31 ±
	10 mg/kg	6.32 ± 3.98	7.12 ± 3.83	7.25 ± 4.79	5.79 ± 3.75	10.19 ± 3.83	10.83 ± 6.57	7.92 ± 4.99
	30 mg/kg	6.20 ± 1.07	4.55 ±1.71	3.79 ± 0.88	2.08 ± 0.57	14.32 ± 4.23	7.42 ± 1.76	7.45 ± 1.23
Total monocytes (10^9/liter)	Control	0.87 ±	0.81 ±	0.74 ±	0.91 ±	1.07 ±	1.10 ±	0.93 ±
	10 mg/kg	0.73 ± 0.27	0.66 ± 0.39	0.41 ± 0.27	0.36 ± 0.15	2.01 ± 2.35	1.41 ± 1.53	0.61 ± 0.35
	30 mg/kg	0.75 ± 0.08	0.78 ± 0.32	0.32 ± 0.14	0.08 ± 0.03	7.96 ± 4.72	1.20 ± 0.48	0.82 ± 0.36
Neutrophil (%)	Control	36.9 ±	38.1 ±	33.5 ±	37.1 ±	36.6 ±	34.4 ±	35.9 ±
	10 mg/kg	47.4 ± 28.2	40.9 ± 4.7	37.3 ± 21.7	22.0 ± 10.4	20.5 ± 25.7	52.4 ± 16.2	40.8 ± 7.7
	30 mg/kg	49.1 ± 20.1	55.1 ± 12.4	34.6 ± 8.2	2.0 ± 1.6	62.5 ± 21.8	46.9 ± 5.7	52.4 ± 0.4
Lymphocyte (%)	Control	55.3 ±	53.0 ±	60.0 ±	54.8 ±	55.1 ±	59.4 ±	55.7 ±
	10 mg/kg	49.4 ± 25.8	54.6 ± 3.8	58.6 ± 20.1	71.2 ± 11.9	71.5 ± 17.2	42.8 ± 14.2	62.6 ± 7.0
	30 mg/kg	45.3 ± 17.9	37.6 ± 12.2	31.9 ± 9.0	94.1 ± 2.5	66.5 ± 5.3	55.7 ± 6.6	63.3 ± 6.4
Monocyte (%)	Control	7.2 ±	6.8 ±	7.1 ±	7.6 ±	7.4 ±	8.2 ±	6.8 ±
	10 mg/kg	3.0 ± 2.4	5.0 ± 2.3	3.4 ± 1.1	5.1 ± 1.1	11.8 ± 10.6	4.6 ± 3.8	5.0 ± 1.7
	30 mg/kg	4.2 ± 1.4	6.4 ± 1.7	2.6 ± 0.6	3.8 ± 1.3	14.6 ± 9.1	7.2 ± 2.3	7.1 ± 3.6
Total erythrocytes (10^{12}/liter)	Control	5.44 ±	4.83 ±	4.69 ±	4.68 ±	4.98 ±	5.03 ±	5.24 ±
	10 mg/kg	5.58 ± 0.34	5.03 ± 0.25	4.72 ± 0.30	4.21 ± 0.26	5.04 ± 0.06	5.06 ± 0.07	4.96 ± 0.16
	30 mg/kg	5.34 ± 0.56	4.93 ± 0.38	4.64 ± 0.6	3.95 ± 0.26	3.59 ± 0.08	4.00 ± 0.12	4.73 ± 0.22
Total reticulocytes (10^9/liter)	Control	61.7 ±	55.7 ±	63.5 ±	67.2 ±	72.0 ±	63.0 ±	71.1 ±
	10 mg/kg	72.8 ± 12.6	66.5 ± 22.2	65.0 ± 28.5	42.0 ± 10.3	379.3 ± 165.6	147.0 ± 66.0	145.7 ±50.6
	30 mg/kg	59.6 ± 6.9	51.1 ± 11.8	23.5 ± 7.5	28.7 ± 12.0	146.1 ± 31.1	289.0 ± 102.3	130.8 ± 61.7

Supplementary Table 3 Effect of H-Zt/g4-MMAE *in vivo* on various enzymatic activities in blood samples collected from cynomolgus monkeys

Measurement of Blood Cells	Treatment with ADC	Dynamic changes of blood Enzymatic levels after H-Zt/g4-MMAE injection (days, Mean ± SD)							Clinical implications
		0	2	4	8	15	22	29	
Alanine transaminase (ALT, Unit/L)	Control	42 ±	43 ±	44 ±	63 ±	52 ±	38 ±	40 ±	Liver damage
	10 mg/kg	57 ± 19	110 ± 39	127 ± 34	86 ± 25	56 ± 13	57 ± 8	53 ± 18	
	30 mg/kg	45 ± 22	79 ± 40	122 ± 67	95 ± 35	40 ± 11	34 ± 9	34 ± 11	
Alkaline phosphatase (ALP, Unit/L)	Control	297 ±	357 ±	367 ±	456 ±	428 ±	362 ±	392 ±	Liver damage or bone disorder
	10 mg/kg	413 ± 53	353 ± 37	384 ± 46	510 ± 22	517 ± 15	462 ± 49	429 ± 31	
	30 mg/kg	352 ± 129	311 ± 96	339 ± 117	486 ± 224	629 ± 357	428 ± 159	444 ± 174	
Aspartate transaminase (AST, Unit/L)	Control	55 ±	62 ±	88 ±	69 ±	41 ±	38 ±	42 ±	Liver or heart damage
	10 mg/kg	46 ± 9	135 ± 65	96 ± 10	75 ± 12	53 ± 3	49 ± 8	42 ± 7	
	30 mg/kg	58 ± 31	255 ±125	394 ± 254	171 ± 98	64 ± 8	51 ± 8	50 ± 12	
Gamma-glutamine transaminase (γ-GT, Unit/L)	Control	54 ±	39 ±	38 ±	42 ±	44 ±	40 ±	44 ±	Liver damage
	10 mg/kg	52 ± 9	50 ± 14	49 ± 16	49 ± 15	48 ± 17	51± 15	52 ± 17	
	30 mg/kg	44 ± 18	42 ± 18	40 ± 19	35 ± 6	44 ± 17	43 ± 19	41 ± 18	
Lactate dehydrogenase (LDH, Unit/L)	Control	342 ±	312 ±	335 ±	425 ±	414 ±	297 ±	350 ±	Various tissue damage
	10 mg/kg	338 ± 85	875 ± 159	745 ± 184	801 ± 290	700 ± 122	373 ± 30	349 ± 104	
	30 mg/kg	412 ± 27	1360 ± 396	1,694 ± 820	1,073 ± 206	1,364 ± 543	727 ± 294	534 ± 170	
Creatine kinase (CPK, Unit/L)	Control	171 ±	156 ±	148 ±	151 ±	171 ±	169 ±	176 ±	Muscle damage
	10 mg/kg	167 ± 27	656 ± 366	1,413 ± 180	268 ± 35	238 ± 29	198 ± 18	174 ± 15	
	30 mg/kg	183 ± 39	530 ± 306	7,734 ± 562	861 ± 565	208 ± 102	169 ± 21	186 ± 35	
